# Identification of New PNEPs Indicates a Substantial Non-PEXEL Exportome and Underpins Common Features in *Plasmodium falciparum* Protein Export

**DOI:** 10.1371/journal.ppat.1003546

**Published:** 2013-08-08

**Authors:** Arlett Heiber, Florian Kruse, Christian Pick, Christof Grüring, Sven Flemming, Alexander Oberli, Hanno Schoeler, Silke Retzlaff, Paolo Mesén-Ramírez, Jan A. Hiss, Madhusudan Kadekoppala, Leonie Hecht, Anthony A. Holder, Tim-Wolf Gilberger, Tobias Spielmann

**Affiliations:** 1 Bernhard Nocht Institute for Tropical Medicine, Parasitology Section, Hamburg, Germany; 2 Institute of Zoology and Zoological Museum, University of Hamburg, Hamburg, Germany; 3 Swiss Federal Institute of Technology (ETH) Zürich, Department of Chemistry and Applied Biosciences, Zurich, Switzerland; 4 Division of Parasitology, MRC National Institute for Medical Research, Mill Hill, London United Kingdom; 5 M.G. DeGroote Institute for Infectious Disease Research and Department of Pathology and Molecular Medicine, McMaster University, Hamilton, Ontario, Canada; Philipps-University Marburg, Germany

## Abstract

Malaria blood stage parasites export a large number of proteins into their host erythrocyte to change it from a container of predominantly hemoglobin optimized for the transport of oxygen into a niche for parasite propagation. To understand this process, it is crucial to know which parasite proteins are exported into the host cell. This has been aided by the PEXEL/HT sequence, a five-residue motif found in many exported proteins, leading to the prediction of the exportome. However, several PEXEL/HT negative exported proteins (PNEPs) indicate that this exportome is incomplete and it remains unknown if and how many further PNEPs exist. Here we report the identification of new PNEPs in the most virulent malaria parasite *Plasmodium falciparum*. This includes proteins with a domain structure deviating from previously known PNEPs and indicates that PNEPs are not a rare exception. Unexpectedly, this included members of the MSP-7 related protein (MSRP) family, suggesting unanticipated functions of MSRPs. Analyzing regions mediating export of selected new PNEPs, we show that the first 20 amino acids of PNEPs without a classical N-terminal signal peptide are sufficient to promote export of a reporter, confirming the concept that this is a shared property of all PNEPs of this type. Moreover, we took advantage of newly found soluble PNEPs to show that this type of exported protein requires unfolding to move from the parasitophorous vacuole (PV) into the host cell. This indicates that soluble PNEPs, like PEXEL/HT proteins, are exported by translocation across the PV membrane (PVM), highlighting protein translocation in the parasite periphery as a general means in protein export of malaria parasites.

## Author Summary

Malaria parasites multiply within erythrocytes, highly specialized cells that require profound alterations to support parasite survival. In order to understand how the parasite takes over the host cell it is necessary to know its molecular toolbox to carry out this process, which consists of the proteins exported from the parasite into the host cell. While many such proteins are known because they contain the clearly defined PEXEL/HT motif, an unknown number of further proteins lack such a motif. Here we present the identification of several of these PEXEL negative exported proteins (PNEPs). We analyzed the sequences mediating export in a subset of these proteins and find that in PNEPs lacking an N-terminal signal peptide, the N-terminal region of these proteins is sufficient to mediate export. Thus, despite the lack of a clear signature sequence, these regions share a property mediating export. In addition, we found evidence that suggests that soluble PNEPs get transported into the host cell by translocation across the parasitophorous membrane that surrounds the parasite. This adds a further group of proteins to share this property and highlights protein translocation as a general means of export in all types of malaria proteins tested so far.

## Introduction

Malaria is a major cause of infection-related deaths worldwide [1]. Proliferation of the parasite in red blood cells (RBCs) is responsible for the manifestations of the disease [2]. Within the erythrocyte the parasite grows in a parasitophorous vacuole (PV) to produce multiple invasive daughter cells that, after egress from the host cell, infect new RBCs to continue the cycle. In each round of multiplication the parasite progresses through distinct morphological phases, the ring-, trophozoite- and schizont stage [3], [4]. The erythrocyte lacks the major histocompatibility complex and a nucleus and therefore provides an immunologically privileged environment for this multiplication phase. However, the uniform composition of this niche also poses challenges, requiring extensive host cell modifications that are mediated by a large number of exported parasite proteins [5]. Knowing the complement of all exported proteins, the ‘exportome’, is a prerequisite to understand this process.

Exported proteins in malaria parasites can be divided into two groups. The first, large and well-defined group consists of proteins containing a short telltale motif termed PEXEL (plasmodium export element) or HT (host targeting signal) that is essential for export [6], [7]. *Plasmodium falciparum*, the causative agent of the severest form of human malaria, was estimated to contain a PEXEL/HT-based exportome of 300–400 proteins [6]–[9]. Of these, approximately 75% are part of protein families, leaving an exportome of ∼100 phylogenetically unrelated exported proteins [8]. PEXEL/HT proteins are believed to be transported along a vesicular pathway to the PV where they are transported by a translocation machine across the surrounding parasitophorous vacuole membrane (PVM) to reach the host cell [10], [11]. In a large-scale gene knock-out study the majority of the *P. falciparum* PEXEL/HT proteins tested were not essential for *in vitro* growth but were found to play a role in trafficking of the major parasite virulence factor *Pf*EMP1 to the host cell surface or to affect the rigidity of infected erythrocytes [12].

Exported proteins of the second group do not contain a PEXEL/HT motif [13]. These PEXEL negative exported proteins (PNEPs) are few and so far have been discovered by chance. They include proteins such as SBP1 [14], MAHRP1 [15], REX1 [16], REX2 [17] and MAHRP2 [18]. These PNEPs share a similar domain organization characterized by the lack of a classical N-terminal signal peptide and the presence of a single internal hydrophobic stretch. They all localize to vesicular cisternae termed ‘Maurer's clefts’ or cleft-associated structures termed ‘tethers’. Maurer's clefts are parasite-induced structures in infected RBCs and are believed to be involved in trafficking of proteins to the host cell surface [5]. Similar to many PEXEL/HT proteins, these PNEPs are not essential for parasite growth *in vitro*, with the possible exception of MAHRP2 for which a gene knock out has not been achieved to date. MAHRP1 and SBP1 are important for trafficking of the virulence factor PfEMP1 [19]–[21] and genetic ablation of REX1 leads to stacking of Maurer's clefts and also affects PfEMP1 trafficking [22], [23]. The function of REX2 is not known but parasites lacking the genomic region encoding both REX1 and REX2 are viable *in vitro* [17], [24].

Despite the lack of a clear-cut export motif in PNEPs [18], [25]–[27], we recently showed that the N-terminal sequences of SBP1, REX1, REX2, MAHRP1 and MAHRP2 were all capable of driving export of a reporter, indicating a unifying principle in the export of these PNEPs [28]. Moreover we found similarities between PNEP and PEXEL/HT export including a need for unfolding of integral transmembrane domain (TM) containing PNEPs, suggesting translocation based delivery into the host cell [28]. In contrast to the export of soluble PEXEL/HT proteins [10], inhibition of unfolding caused an arrest of TM-containing PNEPs at the parasite plasma membrane, not the PVM.

As PNEPs so far cannot be predicted using primary sequence information, it remains unclear if they are rare exceptions or if there are more such proteins. We report here the identification of more than 10 novel PNEPs. Our data indicate that PNEPs are more numerous and of more diverse structure than anticipated, and may make up a sizeable fraction of the *P. falciparum* exportome. Analysis of export domains and mode of delivery into the host cell for a subset of these new PNEPs highlights unifying principles in PNEP export and indicates translocation as the mode of export also for soluble PNEPs.

## Results

### Identification of new PNEPs based on transcription profile

**Figure 1:**
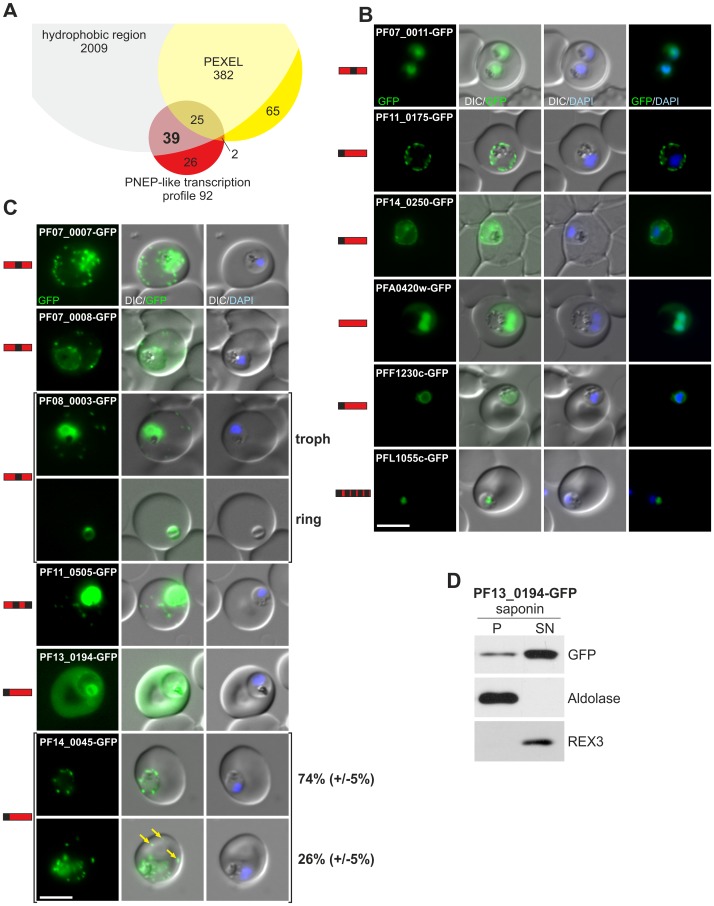
Transcription profile based screen for new PNEPs. (A) Venn diagram of selection of PNEPs based on a transcription profile similar to genes encoding known PNEPs and ETRAMPs. (B, C) Fluorescence pattern of non-exported (B) or exported (C) GFP fusion proteins. Protein structure of each candidate is indicated before each panel (red bars with hydrophobic regions indicated in black, not to scale). For PF08_0003-GFP two panels showing a trophozoite stage (troph) and a ring stage (ring) parasite are shown to demonstrate the different localisations in these stages. For PF14_0045-GFP two panels are shown to demonstrate cells with (yellow arrows) and without additional foci of fluorescence in the host cell (ratio indicated in %, at least 50 cells were analysed on 3 occasions, standard deviation in brackets). Nuclei were stained with DAPI. Size bars: 5 µm. (D) The fluorescence in the host cell of PF13_0194-GFP represents full length soluble protein as determined by Western blot using anti-GFP antibodies with extracts from saponin lysed infected RBCs separated into pellet (P) and supernatant (SN). Parasite cytosolic aldolase was used to control for parasite integrity; REX3 (found soluble in infected RBCs [17]) was used as a control for release of infected host cell cytosol.

Most known PNEPs were highly represented in a small set of genes identified in a ring stage specific cDNA library [29]. We took advantage of this fact to search for new PNEPs. We used ‘Expression Profile Similarity’ in PlasmoDB (version 5.4) to query for genes showing similar transcription profiles to the genes encoding REX1, REX2, SBP1 and MAHRP1 as well as ETRAMP2 and 11.1 (also present in the ring stage-specific library, suggesting a similar transcription pattern to that of PNEPs [29]). For all six query genes the 50 best hits were chosen and pooled, resulting in 92 genes after removal of redundant hits. The high redundancy is consistent with the similar transcription profiles of the query PNEPs. From these 92 genes we then removed all encoding PEXEL proteins (PlasmoDB ExportPred Score of 5 or higher). Of the remaining 65 proteins 39 contained at least one hydrophobic stretch, a feature likely to be present in an exported protein (Figure 1A). Among these 39 proteins (Table S1) were all the PNEPs used in the search due to reciprocal retrieval in the expression profile query. The group also included all 6 ring stage specific members of the *etramp* family [30]. Among the remaining 29 genes, 10 were considered likely false positives (Table S1), for instance Pf*crt* a protein of the food vacuole membrane [31]. The remaining 19 new PNEP candidates were inspected for PEXEL/HT motifs missed by ExportPred to remove another 7 genes (except for MAL13P1.268 all of these were annotated as PEXEL proteins in later versions of PlasmoDB). We also included one of the hits without a hydrophobic region, a protein that contains repeats, which are a feature frequently found in exported proteins. The final set therefore consisted of 13 PNEP candidates (Table S1).

To test whether these proteins were exported, each candidate was tagged with GFP and expressed episomally in *P. falciparum* under the control of the *crt* promoter (a gene with a matching transcription profile as evident from its presence as a false positive in the candidate list). GFP-tagging was at the C-terminus, a position that has not interfered with the location of previously known PNEPs [17], [18], [25], [26], [32]. Western blots showing expression of the GFP fusion proteins for the cell lines generated in this study are shown in Figure S1. Of the cell lines obtained, one showed no detectable GFP fluorescence (PFB0485c-GFP), six showed no evidence of export (Figure 1B) and six showed export (Figure 1C). Of the non-exported proteins PF07_0011-GFP and PFA0420w-GFP were in the nucleus with a cytoplasmic pool, PF11_0175-GFP and PF14_0250-GFP were at the parasite periphery (consistent with a PPM, PV or PVM location and in agreement with the reported location for PF11_0175 [11]), PFF1230c was perinuclear, suggesting an ER location, and PFL1055c was found in a nucleus proximal focus that co-localized with the Golgi marker GRASP [33] by immunofluorescence assay (IFA) (Figure S2A). Of the six exported proteins PF07_0007-GFP, PF07_0008-GFP, PF08_0003-GFP and PF11_0505-GFP produced a punctate appearance in the host cell (Figure 1C) that was confirmed by IFA to correspond to Maurer's clefts (Figure S2B). This agrees with a recent report that showed a host cell location of triple HA tagged PF07_0007 [34]. Cells expressing PF07_0007-GFP and PF07_0008-GFP frequently also showed a uniform fluorescence in the host cell cytoplasm in addition to the staining of Maurer's clefts. In the case of the PF08_0003-GFP cell line, ring stage parasites showed no export and later stages showed only partial export, with additional parasite staining at the nuclear periphery typical of an ER location. PF11_0505-GFP also showed prominent parasite-internal fluorescence in addition to a location at the Maurer's clefts. PF11_0505 is a small protein (89 amino acids) terminating in a second predicted TM; therefore GFP adds a large extra domain that might strongly affect the export efficiency of the chimeric protein. We therefore also generated a parasite line expressing a myc-tagged version of this protein that confirmed the Maurer's clefts location (Figure S3).

Of the remaining two putative exported proteins PF13_0194-GFP was found soluble in the host cell based on the fluorescence pattern and Western blot analysis with selectively lysed infected RBCs (Figure 1C and D). GFP fluorescence was also present in the food vacuole, likely representing protein re-internalized from the host cell cytosol. Some cells also showed some accumulation of the protein at the parasite periphery in addition to the exported fraction (not shown). PF14_0045-GFP showed mobile foci at the parasite periphery and in 26% (+/−5%) of cells one or more foci inside the host cell (Figure 1C, and Figure S4A and B; Video S1 and S2). The foci at the parasite periphery may also represent exported protein. This interpretation is supported by two findings: firstly, Bodipy-TR-C_5_-ceramide staining of parasite membranes indicated that the GFP fluorescence was in close proximity to, but outside of, the parasite periphery (Figure S4A and B); secondly, pre-embedding immuno-EM resulted in a labeling of electron dense areas what appears to be on the outside of the PVM (Figure S4C and D).

Overall the analysis of the candidate set selected on transcription data revealed 5 new PNEPs, and one possible new PNEP (PF14_0045, see discussion). This includes two new types of PNEPs not present in the query set: one with two internal hydrophobic sequences (PF11_0505) and one with a classical N-terminal signal peptide (PF13_0194).

### Identification of novel PNEPs based on chromosomal location of the gene

**Figure 2:**
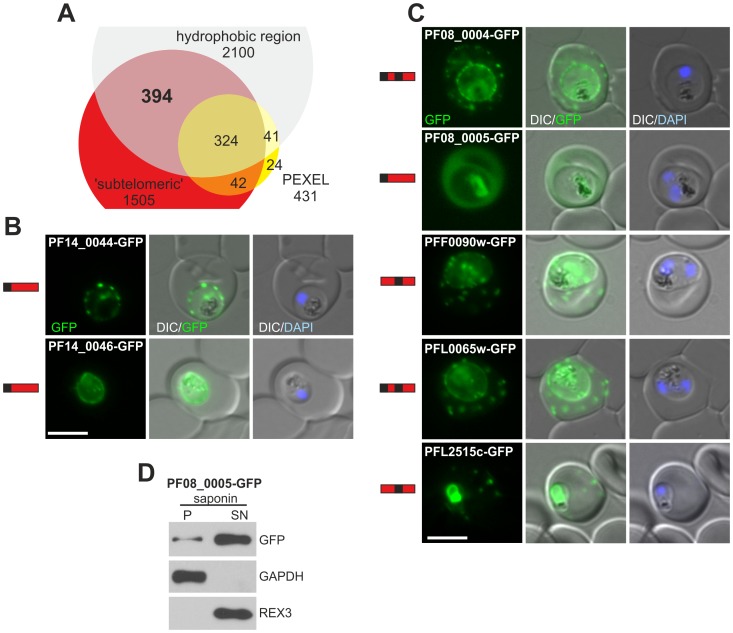
Genomic position based screen for new PNEPs. (A) Venn diagram of selection of PNEPs based on a subtelomeric gene location (<200 kb from chromosome end). (B, C) Fluorescence pattern of non-exported (B) or exported (C) GFP fusion proteins. Protein structure of each candidate is indicated to the left of each panel as in Figure 1. Nuclei were stained with DAPI. Size bars: 5 µm. (D) The fluorescence in the host cell of PF08_0005-GFP represents full length soluble protein as determined by Western blot as described in Figure 1D, except that GAPDH was used as the parasite internal control.

Many of the previously known PNEPs and all but one (PF13_0194) of the novel PNEPs identified here are encoded by subtelomeric genes, consistent with an enrichment of genes for exported proteins in this region of the genome [8]. We therefore carried out a second screen, selecting all genes within 200 kb of the telomeres (1505 genes, PlasmoDB 7.0). Removal of proteins without hydrophobic region and/or with a PEXEL/HT resulted in 394 candidates (Figure 2A). We selected 10 of these candidates for experimental validation of export (Table S2). This included 5 genes from the loci of new PNEPs found in this study and 5 further genes picked arbitrarily from other loci. All selected candidates were of unknown function (according to PlasmoDB annotation), which is typical for many exported proteins [6].

The selected candidate proteins were C-terminally tagged with GFP and expressed in *P. falciparum*. Three GFP fusion proteins (PF07_0010-GFP, PF14_0024-GFP and PFC1035w-GFP) could not be located due to poor fluorescence. Two, PF14_0044-GFP and PF14_0046-GFP, were not exported and showed a fluorescence pattern at the parasite periphery typical of a PPM, PV or PVM location (Figure 2B). The remaining 5 candidates were exported and therefore represent new PNEPs. PF08_0004-GFP, PFF0090w-GFP, PFL0065w-GFP and PFL2515c-GFP showed a punctate pattern in the host cell (Figure 2C) that was confirmed to represent Maurer's clefts (Figure S5). Cells of all of these parasite lines also showed staining at the parasite periphery and in some cases a perinuclear fluorescence indicative of an ER location, in addition to the export. This is most likely due to the tagging with GFP but may also indicate a true dual location for some of these proteins. The fifth exported protein, PF08_0005-GFP, was found soluble in the host cell (again including fluorescence in the food vacuole likely from re-internalized protein) as evident from the fluorescence pattern and from Western blots with selectively lysed infected RBCs (Figure 2C and D).

In conclusion, this approach yielded 5 more PNEPs, including PNEPs of the new type with a classical N-terminal signal peptide (PF08_0005) as well as a further new structural type with an N-terminal signal peptide and a predicted TM region (PF08_0004 and PFL0065w).

### Sequence similarity to the PNEP PF13_0194 uncovers further PNEPs outside of the initial search parameters

PF13_0194 is the only PNEP identified here that is not encoded by a subtelomeric gene. It is found in a locus containing genes coding for MSP7 and MSP7-related proteins (MSRPs) but was not itself considered to be an MSRP [35]. Blast searches with PF13_0194 revealed some similarity to two proteins, PF13_0191 and PF13_0192, found at the same locus but originally (PlasmoDB 5.5) none of these were annotated as MSP7-related (PF13_0191 but not PF13_0192 is now annotated as an MSRP). While PF13_0191 (MSRP5) had a similar structure to the new type of PNEP with a classical N-terminal signal peptide, PF13_0192 is annotated in PlasmoDB with an extra N-terminal exon adding a short sequence before the signal peptide. Inspection of RNAseq data [36] and clones from the Malaria Full Length cDNA database [37] indicated that this exon is not present, and therefore the protein contains a classical N-terminal signal peptide (start ATG at chromosomal position 1,407,512).

**Figure 3:**
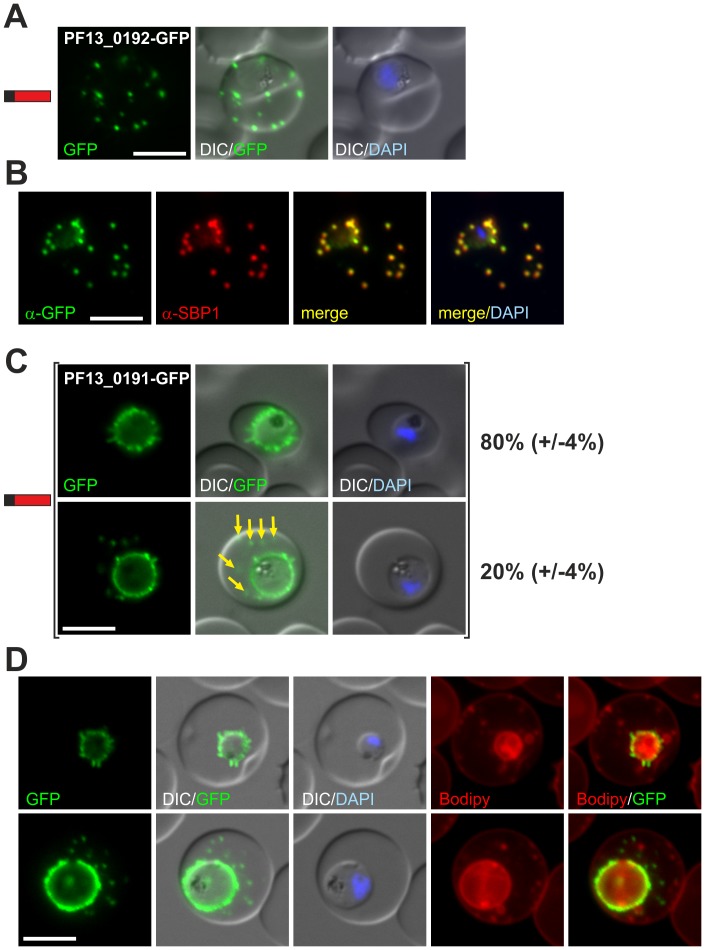
Further PNEPs encoded by genes at the *msp7* gene locus. (A) Fluorescence pattern of PF13_0192-GFP. (B) Co-localisation IFA of PF13_0192-GFP with SBP1. (C) Fluorescence pattern of PF13_0191-GFP. Two panels are shown to demonstrate cells with (yellow arrows) and without additional foci of fluorescence in the host cell (ratio indicated in %, at least 50 cells were analysed on 3 occasions, standard deviation in brackets). (D) Bodipy-TR-C_5_-ceramide (Bodipy) stained parasites expressing PF13_0191-GFP. Protein structure in A and C indicated as in Figure 1. Nuclei were stained with DAPI. Size bars: 5 µm.

The partial similarity of PF13_0191 and PF13_0192 to PF13_0194 prompted us to analyze the location of these proteins in *P. falciparum* by tagging with GFP. PF13_0192-GFP was exported to foci in the host cell (Figure 3A) that were confirmed to be Maurer's clefts (Figure 3B). PF13_0191-GFP localized to foci and mobile protrusions at the parasite periphery and in 20% (+/−4%) of cells was also found in usually multiple mobile foci in the host cell with no apparent contact to the parasite periphery (Figure 3C and Video S3). Co-staining of parasites with Bodipy-TR-C_5_-ceramide showed that the structures protruding from the parasite periphery did not belong to the lipid continuum of the PVM or the tubovesicular network and the foci in the host cell did not co-locate with other structures such as Maurer's clefts that were labeled by Bodipy-TR-C_5_-ceramide (Figure 3D). PF13_0191-GFP therefore appears to reach from the outer face of the PVM into the host cell cytoplasm. Immuno-EM also suggested a location at structures in the host cell (Figure S6). We conclude that PF13_0192 and possibly also PF13_0191 (see discussion) are exported and represent new PNEPs.

### The MSP7 related protein family contains exported proteins

**Figure 4:**
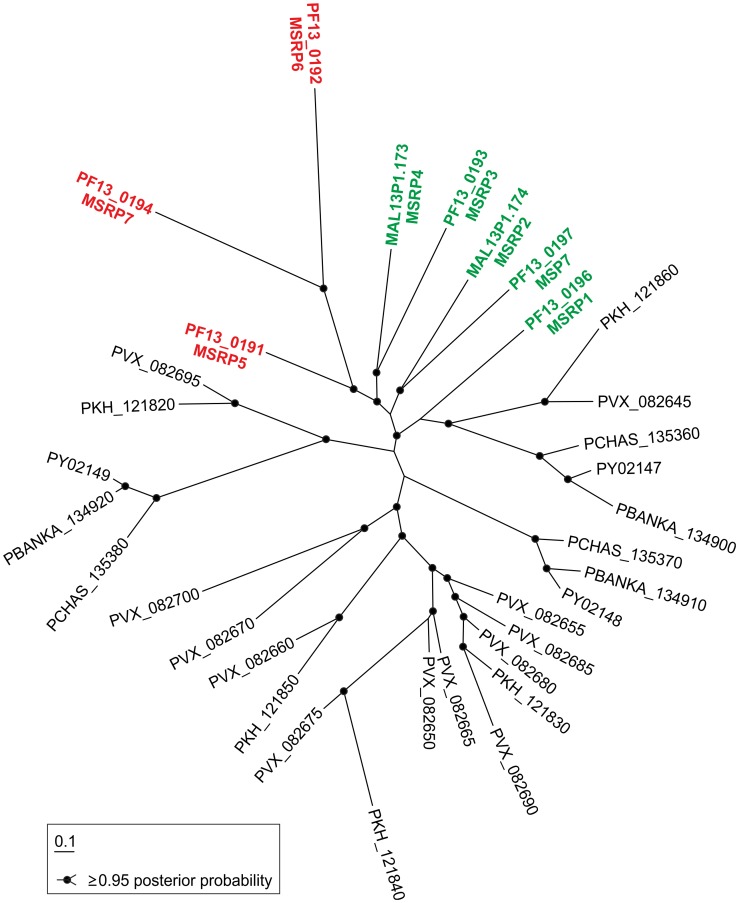
Phylogenetic tree of MSRPs. Phylogenetic tree of 34 MSRPs from different malaria species (indicated by PlasmoDB accession number) retrieved with a HMM profile generated from the available MSP7 and MSRP protein sequences. Note that MSP7 and the seven MSRPs of *P. falciparum* form a monophyletic clade, which also includes five of the 26 MSRPs of other *Plasmodium* species (1.0 posterior probability). Designations of proteins analyzed in this study are shown in red, those of the remaining *P. falciparum* MSRPs in green. Scale bar: 0.1 expected substitution per site.

PF13_0191 was classified as MSRP5 [38] and there was detectable similarity of this protein and PF13_0192 and PF13_0194 with other proteins in the MSRP locus. We therefore analyzed whether these newly found PNEPs are phylogenetically related to the MSRP family. A search with a HMM profile based on the available MSP7 and MSRP sequences from PlasmoDB 9.0 retrieved 34 proteins including PF13_0192 (E-value: 1.9e^−11^) and PF13_0194 (E-value: 4.4e^−10^). Thus, we propose that PF13_0192 and PF13_0194 also belong to the MSRP family and have tentatively named them MSRP6 and MSRP7, respectively. Next, the 34 sequences were aligned and a phylogenetic tree was constructed using a Bayesian approach (Figure 4). The resulting tree shows that today's diversity of the *P. falciparum* MSRPs was largely shaped by several lineage-specific gene duplications and deletions, in agreement with a previously published neighbor-joining tree [35]. Notably, PF13_0191 (MSRP5), PF13_0192 (MSRP6) and PF13_0194 (MSRP7) form a well-supported common clade (0.98 posterior probability) that is most closely related to MSRP3 and MSRP4 of *P. falciparum* (0.98 posterior probability). From these data it can be concluded that the PNEPs found on the MSRP locus share a common evolutionary origin with MSP-7 and MSRPs and emerged by lineage-specific gene duplications from other MSRPs. This analysis suggests unanticipated additional functions in this protein family.

### Characterization of PF13_0192 (MSRP6)

**Figure 5:**
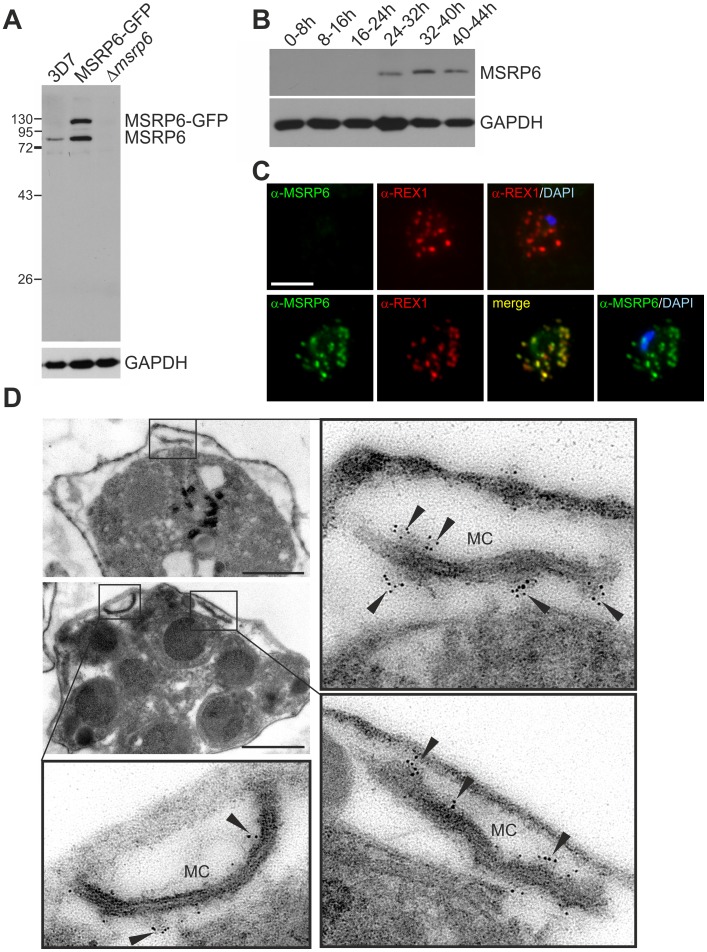
MSRP6 is a Maurer's clefts protein expressed in trophozoites and schizonts. (A) Western blot with parental 3D7 (3D7), MSRP6-GFP expressing parasites and MSRP6 knock out parasites (Δ*msrp6*) demonstrates specificity of the antibodies. Molecular weight standards are indicated in kDa. Parasite GAPDH was used as a loading control. (B) Western blot with specific MSRP6 serum and extracts from stage specific parasites (hours post invasion indicated above each lane). GAPDH was used as a loading control. (C) Co-localisation IFA using anti-REX1 and anti-MSRP6 antibodies with MSRP6 knock out parasites (top) and parental 3D7 parasites (bottom). Size bar: 5 µm. (D) Pre-embedding immuno-EM of infected RBC, where the host cell cytosol has been released with tetanolysin, reacted with anti-MSRP6 antisera. Small panels show overviews with boxes highlighting individual Maurer's clefts for which enlargements are shown. Gold label is indicated with arrowheads. Size bars: 1 µm.

We picked MSRP6 (PF13_0192), a member of the MSRP family and a representative of the new type of PNEP with a classical N-terminal signal peptide for a detailed analysis. We raised specific antibodies against MSRP6 (amino acids 188–320) that recognized a single band of ∼80 kDa in Western blots (Figure 5A). This band was not present in extracts derived from a gene knock out of PF13_0192 targeting the two flanking genes (Δ*msrp3–4*, here termed Δ*msrp6*) [39], demonstrating the specificity of the antibodies. In the PF13_0192-GFP cell line both the endogenous and the transgenic protein were detected (Figure 5A). Western analysis with stage-specific parasite extracts revealed expression of the endogenous MSRP6 from approximately mid cycle (Figure 5B), in agreement with its transcription profile [39]–[41]. Thus, MSRP6, in contrast to most other known PNEPs, is not ring stage-specific.

In IFAs the MSRP6 antiserum recognized foci in infected host cells representing Maurer's clefts as judged by co-localization with REX1 (Figure 5C), in agreement with the results with the MSRP6-GFP fusion (Figure 3A and B). No signal was obtained in Δ*msrp6* parasites (Figure 5C). Using pre-embedding immunoEM with RBCs infected with wild type 3D7 parasites (where the host cell cytoplasm had been released), MSRP6 was detected in ‘cloudy’ structures at the outside of Maurer's clefts (Figure 5D). These structures did not represent the recently described tethers [18], as MSRP6 did not co-localize with the tether marker MAHRP2 (Figure S7A). Close inspection of IFA co-localization with SBP1 and REX1 indicated small differences in localization and staining intensities with respect to MSRP6 that were not present between MSRP6 and MSRP6-GFP (Figure S7A). This might be due to the presence of MSRP6 in the Maurer's clefts associated ‘cloudy’ structures and not the actual Maurer's clefts membrane. This peripheral association of endogenous MSRP6 with the clefts is consistent with the lack of a hydrophobic domain other than the signal peptide in this protein.

No growth or invasion phenotype was previously observed with the Δ*msrp6* parasite line *in vitro* [39]. Checking for defects in trafficking of resident or transient Maurer's clefts or tether proteins we found no change in their location in IFA with Δ*msrp6* parasites (Figure S7B).

### A shared domain promoting export in PNEPs lacking a signal peptide

We previously showed that the N-termini of previously known PNEPs were sufficient to mediate export of a non-exported reporter termed R^REX2TM^ (truncated mTRAP containing a PNEP TM) [27], [28]. This led us to the proposition that this is a general feature of PNEPs. Control mTRAP constructs without PNEP N-terminus or a non-PNEP TM were not exported [28].

**Figure 6:**
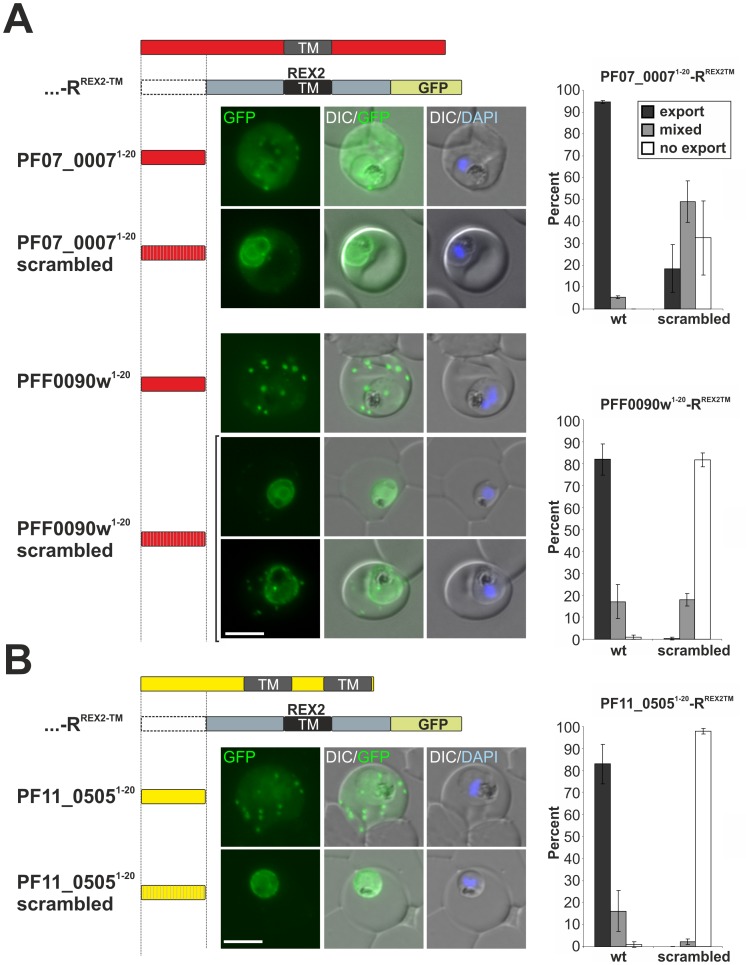
N-termini of new PNEPs without a signal peptide promote export of a reporter with a PNEP TM. (A) Parasites expressing the 20 first amino acids of PF07_0007 (top two rows, wild type and scrambled version of N-termini) or PFF0090w (bottom rows, wild type and scrambled version of N-termini) fused N-terminally to R^REX2-TM^ (truncated mTRAP-GFP containing the REX2TM). For the scrambled PF07_0007 N-terminus the most typical phenotype (mixed) is shown. For the PFF0090w scrambled N-terminus a non-exported and a mixed phenotype is shown. Red bar, PNEP domain; striated bar, randomly scrambled version; grey bar, mTRAP part; green bar, GFP. Export levels are shown to the right for each cell line and were assessed by counting (blinded) the number of cells showing export only (export), export together with parasite periphery and/or internal fluorescence (mixed), or parasite periphery and/or internal fluorescence only (no export). Graphs represent counting of at least 50 cells on three different occasions; error bars represent SD. (B) As in A but parasites expressing R^REX2-TM^ with the first 20 amino acids of PF11_0505 (yellow bar). Nuclei were stained with DAPI. Size bars: 5 µm.

To test whether the newly identified PNEPs of conventional structure (single internal hydrophobic region but lacking a signal peptide) contained similar export information, we fused the first 20 amino acids of two of these (PF07_0007 and PFF0090w) N-terminally to R^REX2TM^ and expressed them in *P. falciparum* (constructs PF07_0007^1–20^-R^REX2TM^ and PFF0090w^1–20^-R^REX2TM^). Both of these constructs were efficiently exported into the host cell (Figure 6A). The staining pattern indicated Maurer's clefts and a soluble pool in the host cell, similar to previous constructs with this reporter [28]. In contrast, reporter containing randomly scrambled versions of these N-termini (PF07_0007^1–20^scrambled-R^REX2TM^ and PFF0090w^1–20^scrambled-R^REX2TM^) showed severely reduced export (Figure 6A). These data indicate that an N-terminal export-promoting domain is a general feature of all PNEPs of this structure.

Of the new types of PNEPs identified here, PF11_0505 contains two predicted TMs but also lacks an N-terminal signal peptide. This prompted us to test whether the first 20 amino acids of PF11_0505 can also mediate export of our reporter (construct PF11_0505^1–20^-R^REX2TM^). Expression in *P. falciparum* resulted in efficient export of this chimera into the host cell (Figure 6B) with a similar pattern to the constructs shown in Figure 6A. Again, a scrambled version of the N-terminus (construct PF11_0505^1–20^scrambled-R^REX2TM^) failed to promote export (Figure 6B). Thus, this new type of PNEP appears to contain similar trafficking information to other PNEPs without an N-terminal signal peptide, suggesting this to be a general principle in the export of these proteins.

### Soluble PNEPs are exported via a translocation step at the PVM

**Figure 7:**
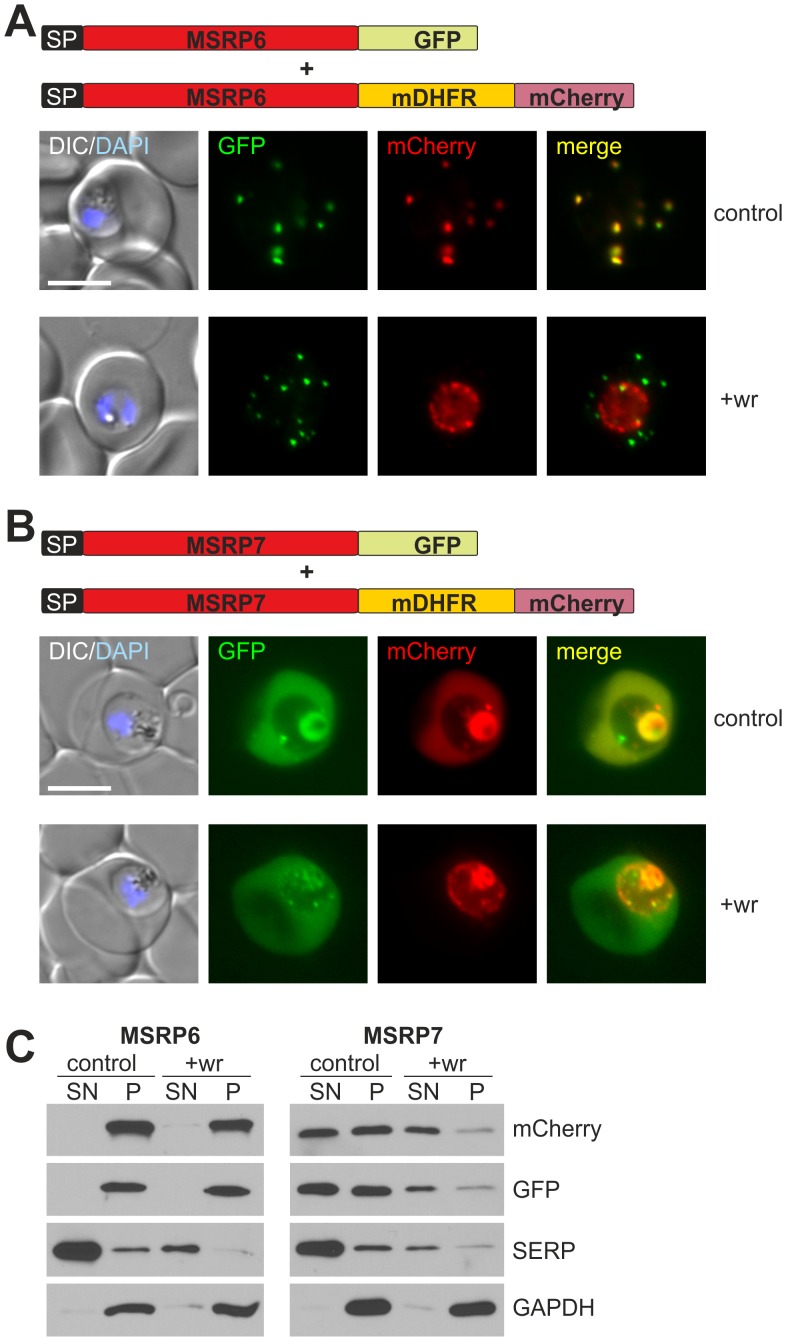
Soluble PNEPs need to be unfolded to reach the host cell. (A) Double transgenic parasites expressing MSRP6-GFP as well as MSRP6 fused to mDHFR and mCherry (constructs indicated on top) grown either with (+wr) or without (control) WR99210. The DIC image with DAPI stained nuclei (DIC/DAPI) and the merged GFP and mCherry signal (merge) are also presented. (B) As in (A) but with parasites expressing MSRP7-GFP and MSRP7-mDHFR-mCherry. The fluorescence internal to the parasite represents re-internalised protein in the food vacuole. Size bars: 5 µm. (C) Western blots of Percoll enriched double transgenic parasites shown in (A) and grown in the absence (control, exported) or presence (+wr, fluorescence remaining in parasite periphery) of WR99210 that were treated with saponin and separated into supernatant (SN) and pellet (P). SERP is found soluble in the PV and was used to demonstrate the action of saponin. GAPDH is the parasite internal control. The intensity of the mCherry, GFP and SERP signals in the extracts of parasites treated with wr is less than that of parasites not treated with wr, which may reflect either a somewhat slower growth cycle or slight differences in the stage composition of the parasite population after sample preparation. MSRP7-mDHFR-mCherry in the PV may also be more efficiently re-internalised and degraded in the food vacuole, reducing its amount in the blocked compared to the unblocked state.

We previously provided evidence that the export of integral TM PNEPs depends on protein translocation [28]. To test whether soluble PNEPs are also exported via a protein translocation step into the host cell, we fused MSRP6 (PF13_0192) and MSRP7 (PF13_0194) (two of the newly identified PNEPs with a classical signal peptide without a TM) with murine dihydrofolate reductase (mDHFR) and mCherry. Unfolding of mDHFR can be prevented with appropriate folate analogs [42]. This system was previously used to show the requirement for unfolding in soluble PEXEL proteins [10]. To have an internal export control for these experiments, we generated double transgenic cell lines: alongside the MSRP-mDHFR-mCherry fusions we expressed the same protein without the mDHFR domain but tagged with a fluorescent protein of different spectral properties (GFP) (Figure 7). In the absence of the folate analogue WR99210, the cell lines expressing MSRP6-mDHFR-mCherry and MSRP7-mDHFR-mCherry both showed export comparable to their internal controls (MSRP6-GFP and MSRP7-GFP, respectively) (Figure 7). However, upon addition of WR99210, the export of the mDHFR-tagged versions was blocked at the parasite periphery whereas the internal controls were still exported (Figure 7). This indicates translocation-based export for these PNEPs.

Release by saponin of parasites containing the export-blocked MSRP7-mDHFR-mCherry showed that the peripheral labeling represented soluble protein (Figure 7C). Sequential lysis of the host cell (with tetanolysin) and the PVM (with saponin) showed that as expected, soluble export-blocked MSRP7-mDHFR-mCherry was present in the PV (Figure S8). This suggests that the block prevents passage through the PVM. MSRP6-mDHFR-mCherry blocked in the parasite periphery remained insoluble, suggesting that this protein aggregated upon accumulation in the PV, which may reflect its propensity to accumulate in the observed ‘cloudy’ structures at the Maurer's clefts membrane (Figure 7C).

## Discussion

Exported proteins enable the malaria parasite to change the host cell into a suitable niche that supports intracellular proliferation, leading to a massive parasite multiplication in the host. To understand this process it is essential to know which parasite proteins are exported. Identification of the PEXEL/HT motif greatly aided the search for exported proteins, leading to a large set of proteins generally referred to as the exportome [6]–[9]. With only few PNEPs known so far [13], these proteins had to be considered an exception to the rule. However, in contrast to the well-defined motif in PEXEL proteins, the lack of a simple signature sequence has prevented a systematic prediction of PNEPs. Consequently the PNEPs known so far were discovered by chance and it remained unknown whether further PNEPs exist and what the magnitude of the PNEP portion of the exportome might be.

Here, using two different selection procedures, we significantly expanded the number of known PNEPs. Our selection criteria, for lack of better options, were rather general. Thus, the list of new PNEPs provided here is likely to be far from comprehensive. Despite these limitations, these approaches yielded a considerable number of new PNEPs, indicating that many more are hidden in the *P. falciparum* genome. It should be noted however that the transcription-based list was exhausted (with all likely candidates tested) and that the subtelomere list of 394 candidates contains many likely false positives (such as proteins of predicted function unlikely to be exported, which were not tested). Nevertheless this second list still contains over 100 proteins of unknown function that were not tested, but which likely includes more PNEPs. It therefore seems conceivable that in total the *P. falciparum* genome encodes several tens of PNEPs. The *P. falciparum* PEXEL/HT exportome was estimated to consist of 109 unique proteins (removing protein families) [8]. Therefore, although it is at present impossible to give a more clearly defined number, PNEPs may make up a sizeable fraction of the exportome. Potentially, this fraction may be even larger in other malaria species that appear to harbor fewer PEXEL/HT proteins than *P. falciparum* [8]. Indeed, several PNEPs have recently been reported from the rodent malaria parasite *P. berghei* although some belong to protein families [43] and only definitive knowledge about the number of unique PNEPs in *P. falciparum* and other malaria species will allow one to judge whether these proteins are underrepresented in *P. falciparum*.

Our data not only suggest a larger number of PNEPs in *P. falciparum* than anticipated but also identify PNEPs of new overall structure. Most previously known PNEPs contain a single internal hydrophobic region [13]. Here we also found PNEPs with a classical N-terminal signal peptide, both with and without an additional TM (Table S3). Additionally, one new PNEP contained 2 internal hydrophobic stretches but no classical signal peptide. A recent report also identified a PNEP with a classical N-terminal signal peptide [44]. Together with our data this indicates that this may be a common structure of exported proteins, despite the initial paucity of such PNEPs. No multi-spanning TM PNEPs with more than 2 TMs were found here, but such proteins were very rare in the two data sets examined; in the first dataset (PNEP transcription profile) only one candidate of this type was found and from the second set (subtelomeric gene location) no such protein was selected. It is therefore possible that such PNEPs exist, although their absence in the first set may indicate that they are uncommon or absent.

The use of GFP-tagging may have reduced the export efficiency of some proteins, leading to partial retention within the parasite as seen with several of the proteins tested here. Alternatively, some of these proteins might naturally be located in more than one compartment. While we consider export as a GFP fusion as a definite marker of protein export (confirmed here by antisera for MSRP6 and for several previously described PNEPs [17], [18], [25], [26], [32]), some questions arose for two of the GFP-tagged proteins from our screen. PF13_0191 (MSRP5) and PF14_0045 showed a new localization represented by accumulation on what appears to be the outside of the PVM. It is possible that this is an overexpression phenotype, although it is unclear how this could explain the presence of individual foci of fluorescence in ∼20% of host cells. Using a serum raised against MSRP5 we detected MSRP5-GFP on Western blots of asexual blood stage extracts but not endogenous MSRP5 (data not shown). A previous study also failed to detect MSRP5 despite clear evidence for transcription of the corresponding gene [39]. This indicates that either MSRP5 is not expressed in these parasites or its expression is below the detection limit (*crt*-promoter driven expression of MSRP5-GFP may be both higher and earlier in the cycle, making possible a detection). In the case of PF14_0045 it should also be noted that this was the only exported candidate truncated due to size (see Materials & Methods). In the absence of a confirmed location for endogenous MSRP5 (PF13_0191) and PF14_0045 (attempts to raise specific antiserum to this protein have so far been unsuccessful, data not shown) for example by immuno-localization with specific antisera, and with GFP-fusion proteins providing the only evidence of a new type of localization, further evidence is required before it can be established with certainty that these are true PNEPs.

A striking finding of this study was that the MSP7 related protein family contains exported proteins. This was supported by both GFP tagging approaches and by analysis of the export of endogenous MSRP6. To date the MSRPs were considered to be important in invasion, which was mostly based on the location of MSP7 in the MSP1 complex on the merozoite surface [35]. However, our data indicates that it is appropriate to think of the MSRP family in a wider context. The MSRPs analyzed here were found to be soluble in the host cell (MSRP7), attached to the Maurer's clefts (MSRP6) and possibly bound to the outside of the PVM (MSRP5). MSRP2 is present in the PV [39]. As two of the three proteins analyzed here are attached to membranes despite lacking a TM, it is possible that by analogy to MSP7 [45], they associate with their target structures via protein-protein interactions. MSRP homology may be due to a shared protein interaction domain that functions in diverse complexes. However, there is little evidence so far for a function of MSRPs. With the exception of MSP7 itself, for which a mild growth phenotype associated with the gene knock out was described [46], ablation of *msrp* genes had no effect on parasite growth *in vitro* [39]. The different locations of different family members make redundancy as an explanation for this unlikely (although not all members have been localized). It is possible therefore that these proteins only have a role *in vivo*. For example, an effect on pathology was ascribed to *P. berghei* MSP7 in a rat disease model, which suggests an immune modulatory role [47]. Other possible roles would need to be evaluated *in vivo* although this will be difficult to do because the MSP7 family is highly heterogeneous between parasite species ([35] and Figure 4).

The large diversity of PNEPs revealed here raises the question of whether these proteins share common export sequences. The biggest group of PNEPs contains a single internal hydrophobic region but no signal peptide. Recently we showed that the N-terminal sequences of the previously established PNEPs with this structure were capable of promoting export of a reporter (truncated mTRAP containing a PNEP TM), suggesting a unifying principle in the export of these PNEPs [28]. Using the same reporter, we show here that the 20 N-terminal amino acids of two new PNEPs of this structure also promote export. In addition we found that the N-terminus of PF11_0505, a new type of PNEP with 2 hydrophobic regions, was also sufficient to export the reporter. Thus, it seems likely that this is a property shared by all PNEPs lacking a classical N-terminal signal peptide, as this so far holds true for 8 out of 8 tested proteins. A role for the N-terminal region was recently also shown for SURFIN 4.1 [48]. However, there is little obvious primary sequence similarity between these N-terminal regions and therefore little opportunity to predict PNEPs *in silico* using the sequence information. The type of TM was previously found to also play a role in export [26]–[28] and it remains to be tested whether this is also the case for the newly discovered PNEPs. While some PNEPs with an internal hydrophobic region are integral membrane proteins [14], [15], [17], REX1 [32] and MAHRP2 are not [18]. It may therefore be possible that some of the newly discovered PNEPs with a single internal hydrophobic region are not integral membrane proteins.

We previously showed that unfolding is needed for the export of PNEPs containing a TM [28]. This indicated that similar to the export of soluble PEXEL/HT proteins [10], [11], TM PNEPs reach the host cell by protein translocation. The PNEPs with new structure discovered here provided the opportunity to test whether this is also the case for soluble PNEPs. Ligand-induced prevention of unfolding blocked export, indicating an involvement of protein translocation in the export of soluble PNEPs. Thus all types of proteins analyzed to date require translocation to reach the host cell. Blocked MSRP7-mDHFR-mCherry was found in the PV, indicating a failure of passage through the PVM. Our previous work on TM PNEPs found a block at the PPM, whereas soluble PEXEL/HT proteins were found in the PV [28]. Thus MSRP7 behaves like soluble PEXEL/HT proteins. Importantly, the 2 known sites of mDHFR folding-induced block in export correlate with membrane association rather than the type of protein, i.e. soluble PEXEL/HT proteins and soluble PNEPs are found in the PV, whereas PNEP TM proteins are found at the PPM. Presumably TM proteins would then pass the PVM in a second translocation step. This would then lead to direct release of the protein into the host cell in a non-membrane bound form, in agreement with the failure to detect vesicular trafficking of TM proteins from the PVM to the Maurer's clefts [4]. PEXEL TM proteins have not been tested so far but blocked mTRAP-mDHFR reporter constructs with a mature PEXEL N-terminus were also retained in the PPM [28], indicating that PEXEL TM proteins might behave like PNEP TM proteins. These data are consistent with translocation events at the parasite periphery as a general principle for exported proteins to reach the host cell, and further highlight similarities in the trafficking pathways of PNEPs and PEXEL proteins. Whether the actual translocation machineries are the same for each type of protein remains to be determined.

## Materials and Methods

### Animal ethics statement

All handling and immunizations of mice were carried out by Eurogentec, Belgium in accordance with good animal practices according to the Belgian national animal welfare regulations for Eurogentec SA, Seraing. Eurogentec had approval (CE/Sante/E/001) from the ethics committee of the Centre d'Economie Rurale (CER Groupe, Marloie, Belgium).

### Parasite culture and transfection

*Plasmodium falciparum* 3D7 parasites were cultured in human 0^+^ erythrocytes and RPMI 1640 medium containing 0.5% AlbuMAX (Invitrogen) according to standard methods [49]. Synchronized parasites were transfected with 100 µg of purified plasmid DNA (Qiagen) as described previously [50]. Positive selection was done with 4 nM WR99210 (Jacobus Pharmaceuticals) or 2 µg/ml Blasticidin S (Invitrogen).

### Plasmid constructs

All inserts were amplified by PCR with Phusion polymerase (NEB) using the primers listed in Table S4. The following genes were truncated due to size or repeats: PF14_0045 (bp1-1506 of 2895), PFA0420w (bp1-432 of 580, repeats), PF07_0010 (bp1-1454 of 5532), and PF14_0250 (bp1-813 of 3963, repeats). For C-terminal GFP fusion of candidate genes expressed under the *crt* promoter, inserts were digested with KpnI/AvrII and cloned into pARL1a^−^GFP [33], [51]. The myc-tagged version of PF11_0505 was cloned into the same vector using KpnI/XhoI to remove the GFP. To add the wildtype or scrambled N-termini (first 20 aa) of PF07_0007, PF11_0505 and PFF0090w to the truncated mTRAP containing the REX2 TM (R^REX2-TM^), inserts were PCR amplified using R^REX2-TM^ [28] as a template and similarly cloned into pARL1a^−^GFP. Scrambled sequences were generated using EMBOSS 6.3.1: shuffleseq (http://mobyle.pasteur.fr/cgi-bin/portal.py?#forms::shuffleseq) leading to: PF07_0007^1–20^scrambled (MANNTQTEAQPQQKAEAGQS), PF11_0505^1–20^scrambled (MKEEETLVEKKKEKAMQKSK) and PFF0090w^1–20^scrambled (MLSFTHLHMGLQNHDDNYLL). mDHFR fusion constructs were generated in pARL2-DG [10] containing blasticidin deaminase as a resistance marker. GFP was exchanged with mCherry using KpnI/XmaI to obtain pARL2-mDHFR-mCherry^BSD^ into which MSRP6 (PF13_0192) and MSRP7 (PF13_0194) inserts were cloned using XhoI/AvrII. For the GST fusion of PF13_0192 (aa 188–320, start ATG chromosomal position 1,407,512), the insert was digested with BamHI/XhoI and cloned into pGEX-6P-2 (GE Healthcare). All constructs were sequenced to exclude clones with mutations.

### Live cell imaging and immunofluorescence assays (IFAs)

Staining of nuclei with 1 µg/ml DAPI (Roche) and of live parasites with 5 µM Bodipy-TR-C_5_-ceramide (Invitrogen) was performed as described [52]. For immunofluorescence assays parasites were dried on 10-well slides and fixed in 100% acetone for 30 min at room temperature. Antibodies were added for 1 h in 3% BSA/PBS in the following dilutions: mouse anti-GFP (Roche): 1/500, rabbit anti-GFP (Open Biosystems): 1/500, mouse anti-GRASP: 1/5000, rabbit anti-KAHRP: 1/500, rabbit anti-MAHRP2: 1/250, rabbit anti-myc (Cell Signaling Technology): 1/250, mouse anti-PF13_0192 (MSRP6): 1/500, rabbit anti-REX1: 1/5000, mouse anti-REX2: 1/1000, rabbit anti-SBP1: 1/375. Secondary antibodies were Alexa Fluor-488 and -594 donkey anti-rabbit and Alexa Fluor-488 and -594 goat anti-mouse (Invitrogen) diluted 1/2000. After each antibody incubation wells were washed with PBS. Parasites were imaged with a Zeiss Axioskop M1 microscope using a 100×/1.4 oil immersion lens. Pictures were taken with a Hamamatsu Orca C4742-95 camera and Zeiss Axiovision software and processed with Corel Photo-Paint X4.

For time-lapse movies cells were imaged with an Olympus FV1000 confocal microscope using an 100×/1.4 oil immersion lens. The Fluoview software v1.7b was used for image collection. The 488 nm laser line was used to excite GFP and to obtain DIC images. Movies were generated in Imaris 6.2.0. and time stamped in Image J (http://rsb.info.nih.gov/ij/).

### Preparation of parasite protein extracts and Western blot analysis

To obtain protein extracts, parasites were released from RBCs using 0.03% saponin/PBS for 20 min on ice, washed in PBS and resuspended in 4% SDS/0.5% TX-114/0.5× PBS. For saponin supernatants, infected RBCs were purified using a Percoll gradient [53], washed in PBS and lysed with 0.03% saponin/PBS for 20 min on ice. After centrifugation for 5 min at 16,000 *g*, equal amounts of supernatant and pellet were used for SDS-PAGE. Selective release of the host cell cytosol and the PV content were done as described previously using Percoll purified parasites that were sequentially treated with 1 U/ml tetanolysin (Sigma) and 0.015% saponin [28] with the modification that 0.2% (w/v) of BSA was added for the tetanolysin lysis. Western blots were performed on nitrocellulose membranes (Schleicher & Schüll) with 10 mM CAPS pH 11.2 transfer buffer in a tank blot device (BioRad). Antibodies were applied in 5% milk/PBS and detection was performed using ECL (GE Healthcare). Antibodies were used in the following dilutions: rabbit anti-aldolase: 1/2000, mouse anti-GAPDH: 1/2000, mouse anti-GFP (Roche) 1/1000, rat anti-mCherry (Chromotek): 1/5000, rabbit anti-myc (Cell Signaling Technology): 1/250, mouse anti-PF13_0192 (MSRP6): 1/500, mouse anti-REX3: 1/2000, rabbit anti-SERP: 1/2000. Secondary antibodies were horseradish peroxidase-conjugated goat anti-mouse (Roche) and goat anti-rat (Dianova), both diluted 1/3000, and donkey anti-rabbit (Dianova) used at 1/2500.

### Expression of recombinant proteins and immunization

GST fusion proteins were expressed in *E. coli* BL21 cells and purified using glutathione-Sepharose (Genscript). Mice were immunized commercially with four injections of 15 µg recombinant protein (carried out by Eurogentec according to their standard procedures).

### Immunoelectron microscopy

For pre-embedding samples of PF13_0191-GFP and PF14_0045-GFP expressing parasites, infected RBCs were Percoll purified, washed with PBS and fixed in 2% formaldehyde for 10 min. After centrifugation for 3 min at 300 *g* the samples were washed and treated with 1 U/ml Tetanolysin (List biological laboratories) in 800 µl PBS for 30 min at 37°C. After centrifugation and washing in PBS the samples were fixed again with 2% formaldehyde for 5 min followed by centrifugation and washing. The parasites were blocked with 3% BSA/PBS for 15 min, and then mouse anti-GFP (Roche) antibodies were applied for 1.5 h in a 1/20 dilution in 3% BSA/PBS. After washing the samples were incubated for 1.5 h with rabbit anti-mouse antibodies (Dako) diluted 1/25 in 3% BSA/PBS. Pellets were washed and resuspended in a 1/20 dilution of Protein A-gold (6 nm, Aurion) in 3% BSA/PBS for 1.5 h. Finally, the samples were washed once in PBS and re-fixed in 0.1 M sodium cacodylate containing 2% glutaraldehyde. All samples were processed for electron microscopy by routine techniques. This involved postfixation in 1% osmium tetroxide for 30–60 min at 4°C, sedimentation in 3% LM Agarose, dehydration in ethanol (30%, 50%, 70%, 80%, 90% and 100% 3×) for 10 min each, treatment with propylene oxide twice for 5–10 min, and embedding in Epon epoxy resin. Ultra thin sections were prepared using an Ultra-Microtome (Ultracut E, Reichert-Jung) with an Ultra-Diamond and stained with 2% uranyl acetate for 5 min and lead citrate for 5 min before examination in a transmission electron microscope (FEI Tecani) at 80 kV.

### Phylogenetic analysis of MSRPs

A profile Hidden Markov Model (HMM) search was conducted employing the HMMER3 package [54]. The HMM profile was generated using MSP7 and the MSRPs from various *Plasmodium* species annotated in PlasmoDB 9.0 [55] and used to identify additional MSRPs. Amino acid sequences with an *E* value below 1.0e-10 were aligned with MAFFT 6.0 using the E-INS-i routine [56]. Phylogenetic reconstructions were performed with MrBayes 3.2 using the GTR model of amino acid evolution with gamma-distributed rate heterogeneity and a proportion of invariant sites [57]. Metropolis-coupled Markov chain Monte Carlo sampling was performed with one cold and three heated chains. Two independent runs were performed for 2,000,000 generations and trees were sampled every 100th generation. Posterior probabilities and convergence of both runs were estimated on the final 15,000 trees (burn-in = 5,000).

## Supporting Information

Figure S1 **Western blots of extracts from the cell lines used in this study.** Origins of the individual extracts are indicated above each blot. The molecular weights of the protein marker bands are indicated in kDa on the first blot. Only some of the bands are labelled in the following blots. For orientation, the 72 kDa marker band is shown as a bold line. For PF13_0194 and PF08_0005 that are found soluble in the host cell the saponin supernatant (SN) and pellet (P) fraction of Percoll enriched parasites are shown. All other extracts were derived from saponin-released parasites. Asterisks indicate degradation products of the fusion protein, likely representing GFP alone.
(TIF)

Figure S2 **Co-localisation IFAs of GFP-tagged proteins from the transcription-based screen.** (A) PFL1055c-GFP detected with anti-GFP antibodies (red) co-locates with the Golgi marker GRASP (green). (B) The GFP-fusion proteins indicated on the right and detected with anti-GFP antibodies (green) co-locate with the Maurer's clefts Marker REX1 (red). DAPI (blue) was used to stain nuclei. Size bars: 5 µm.
(TIF)

Figure S3 **Export of a myc-tagged version of PF11_0505 to the Maurer's clefts.** (A) IFA using anti-myc (red) and the Maurer's clefts marker REX2 (green) show co-location of PF11_0505myc and REX2 at the clefts. DAPI (blue) was used to stain nuclei. Size bars 5 µm. (B) Western blot probed with an anti-myc serum detects PF11_0505myc in the corresponding parasite line but not in 3D7 (filled arrowhead). The band apparent in both parasite extracts may be non-specific luminescence derived from left over hemoglobin (open arrowhead). The molecular weight standard is indicated as described in the legend to Figure S1.
(TIF)

Figure S4 **Localization of PF14_0045-GFP.** (A) Fluorescence of PF14_0045-GFP (green) in Bodipy-TR-C_5_-ceramide (Bodipy, red) stained cells. Top panel cell showing GFP foci at the parasite periphery and bottom row a cell showing an additional focus in the host cell (white arrows). (B) Top, enlargement of the white frame from the top panel in (A) shows that the foci are parasite proximal but do not overlap and are located towards the host cell cytosol if compared to the Bodipy-TR-C_5_-ceramide staining. Bottom, the Bodipy-TR-C_5_-ceramide staining in the last image of the bottom row in (A) was intensified to demonstrate that the PF14_0045-GFP-derived focus in the host cell (white arrow) does not overlap with structures typically stained with Bodipy-TR-C_5_-ceramide such as Maurer's clefts. DAPI (blue) was used to stain nuclei. Size bars: 5 µm. (C) Pre-embedding immuno-EM (host cell cytosol released with Tetanolysin) using gold conjugated anti-GFP antibodies on PF14_0045-GFP expressing parasites. Size bar: 1 µm. The frame is enlarged in (D) and shows accumulation of gold in an electron dense area (blue arrow) that appears to be on the outside of the PVM. RBM (red blood cell membrane) and PVM are indicated.
(TIF)

Figure S5 **Co-localisation IFAs of GFP-tagged proteins from the genetic locus-based screen.** The GFP-fusion proteins indicated on the right and detected with anti-GFP antibodies (green) co-locate with the Maurer's clefts Marker REX1 (red). DAPI (blue) was used to stain nuclei. Size bars: 5 µm.
(TIF)

Figure S6 **Immuno-EM analyis of PF13_0191-GFP expressing parasites.** Pre-embedding immuno-EM (host cell cytosol released with Tetanolysin) using gold conjugated anti-GFP antibodies on PF13_0191-GFP expressing parasites. Size bars: 1 µm. The image to the left shows an enlargement of the region highlighted by a frame in the overview image. Accumulations of gold label in the host cell are indicated by blue arrows. RBM (red blood cell membrane) and PVM are indicated.
(TIF)

Figure S7 **Co-localisation IFAs in 3D7 and MSRP6 knock out parasites.** (A) IFAs with 3D7 parasites with the antisera indicated on the panels. The white frames highlight individual Maurer's clefts in the enlargements shown to the right. (B) IFAs with MSRP6 knock out parasites show no apparent change in the staining pattern typically obtained with the antisera indicated. DAPI (blue) was used to stain nuclei. Size bars 5 µm.
(TIF)

Figure S8 **Export-blocked MSRP7-mDHFR-mCherry can be found soluble in the PV.** Western blots of Percoll enriched double transgenic parasites expressing MSRP7-mDHFR-mCherry and MSRP7-GFP grown in the absence (control) or presence (+wr) of WR99210, treated sequentially with tetanolysin and saponin and separated into supernatant (SN) and pellet (P). REX3 is a parasite protein found in the host cell cytosol and was used as a control for host cell membrane lysis and release of this fraction. SERP is found soluble in the PV and was used to demonstrate the action of saponin. Note that not all of the SERP was released and hence the release of the export-blocked MSRP7-mDHFR-mCherry is equally incomplete. The presence of MSRP7-mDHFR-mCherry over MSRP7-GFP in the PV is highlighted by arrows. The distortion of the MSRP7-mDHFR-mCherry signal in the exported fraction is due to co-migration with BSA used in the tetanolysin lysis. The lower intensity of the mCherry, GFP and SERP signals in the extracts of parasites treated with wr may reflect either a slower growth cycle or differences in the stage composition of the parasite population after sample preparation.
(TIF)

Table S1 **39 candidates with a similar transcription profile to known PNEPs.** Yellow: selected candidates, green: ETRAMPs, blue: known PNEPs, grey: false-positives, orange: newly annotated PEXEL-proteins.
(DOC)

Table S2 **397 candidates with a subtelomeric gene locus.**
(DOC)

Table S3 **Candidates selected for GFP-tagging.**
(DOC)

Table S4 **Primers used in this study.**
(DOC)

Video S1 **Timelapse imaging of a parasite expressing PF14_0045-GFP.** Overview of an infected red blood cell showing a DIC and GFP overlay of single z-sections acquired using the 488 nm laser line (10 frames over 27 seconds). The black box indicates the area of the section shown in Video S2.
(MOV)

Video S2 **Timelapse imaging of a section of the cell shown in Video S1.** The video shows 200 frames over 35 seconds. Left, GFP signal; right, overlay of GFP and DIC. For the left image a gauss filter was used with the filter width suggested by the Imaris software.
(MOV)

Video S3 **Timelapse imaging of a parasite expressing PF13_0191-GFP.** Fifty single z-section were obtained over 16 seconds. Left, GFP signal; right, overlay of GFP and DIC.
(MOV)
