# Supplementary figures and images for: Identification of New PNEPs Indicates a Substantial Non-PEXEL Exportome and Underpins Common Features in Plasmodium falciparum Protein Export
Source: PLoS Pathog. 2013 Aug 8;9(8):e1003546. doi: 10.1371/journal.ppat.1003546 (PMC3738491; doi:10.1371/journal.ppat.1003546)

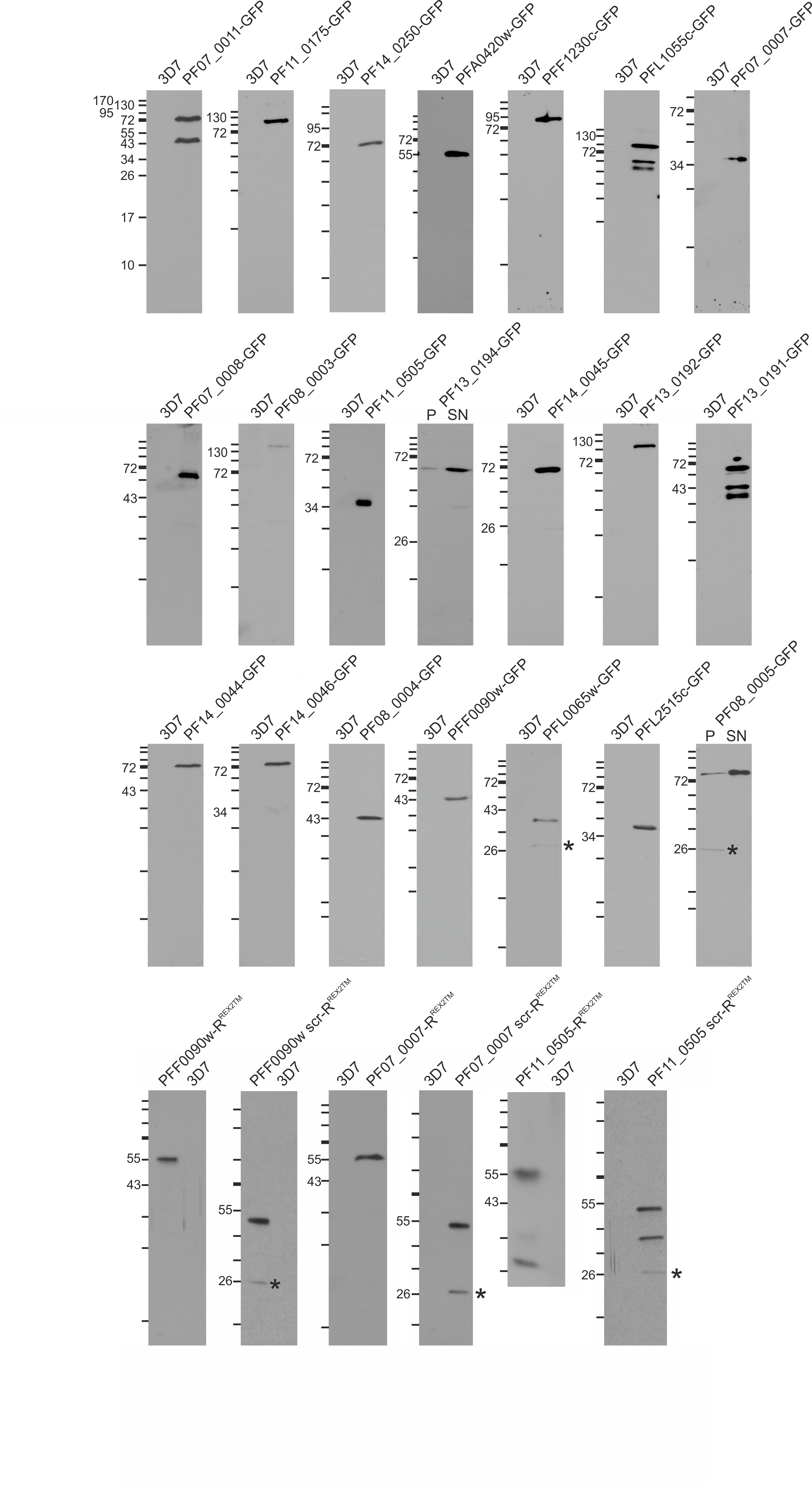

Supplement: Figure S1 — Western blots of extracts from the cell lines used in this study. Origins of the individual extracts are indicated above each blot. The molecular weights of the protein marker bands are indicated in kDa on the first blot. Only some of the bands are labelled in the following blots. For orientation, the 72 kDa marker band is shown as a bold line. For PF13_0194 and PF08_0005 that are found soluble in the host cell the saponin supernatant (SN) and pellet (P) fraction of Percoll enriched parasites are shown. All other extracts were derived from saponin-released parasites. Asterisks indicate degradation products of the fusion protein, likely representing GFP alone. (TIF) [file ppat.1003546.s001.tif]

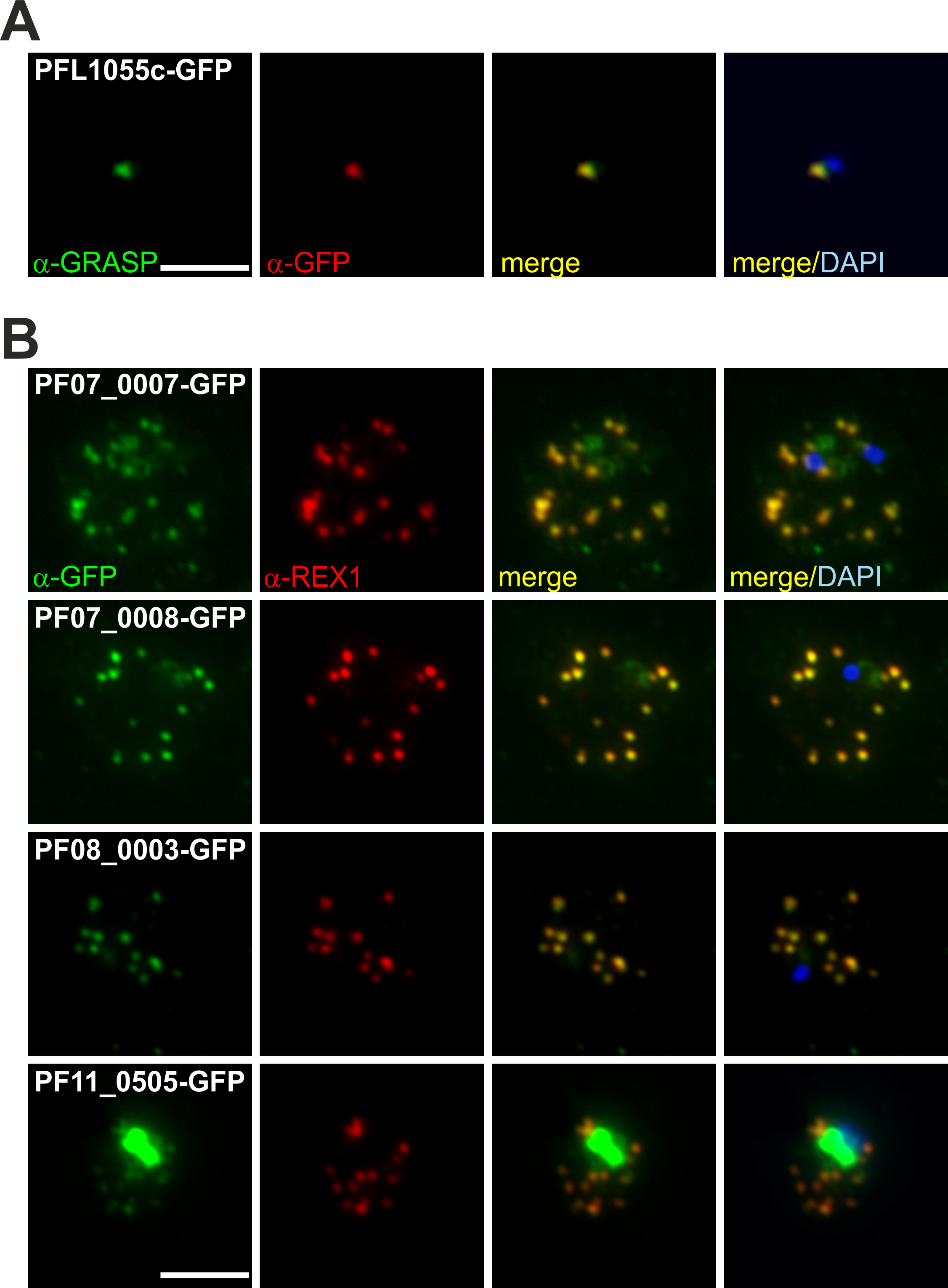

Supplement: Figure S2 — Co-localisation IFAs of GFP-tagged proteins from the transcription-based screen. (A) PFL1055c-GFP detected with anti-GFP antibodies (red) co-locates with the Golgi marker GRASP (green). (B) The GFP-fusion proteins indicated on the right and detected with anti-GFP antibodies (green) co-locate with the Maurer's clefts Marker REX1 (red). DAPI (blue) was used to stain nuclei. Size bars: 5 µm. (TIF) [file ppat.1003546.s002.tif]

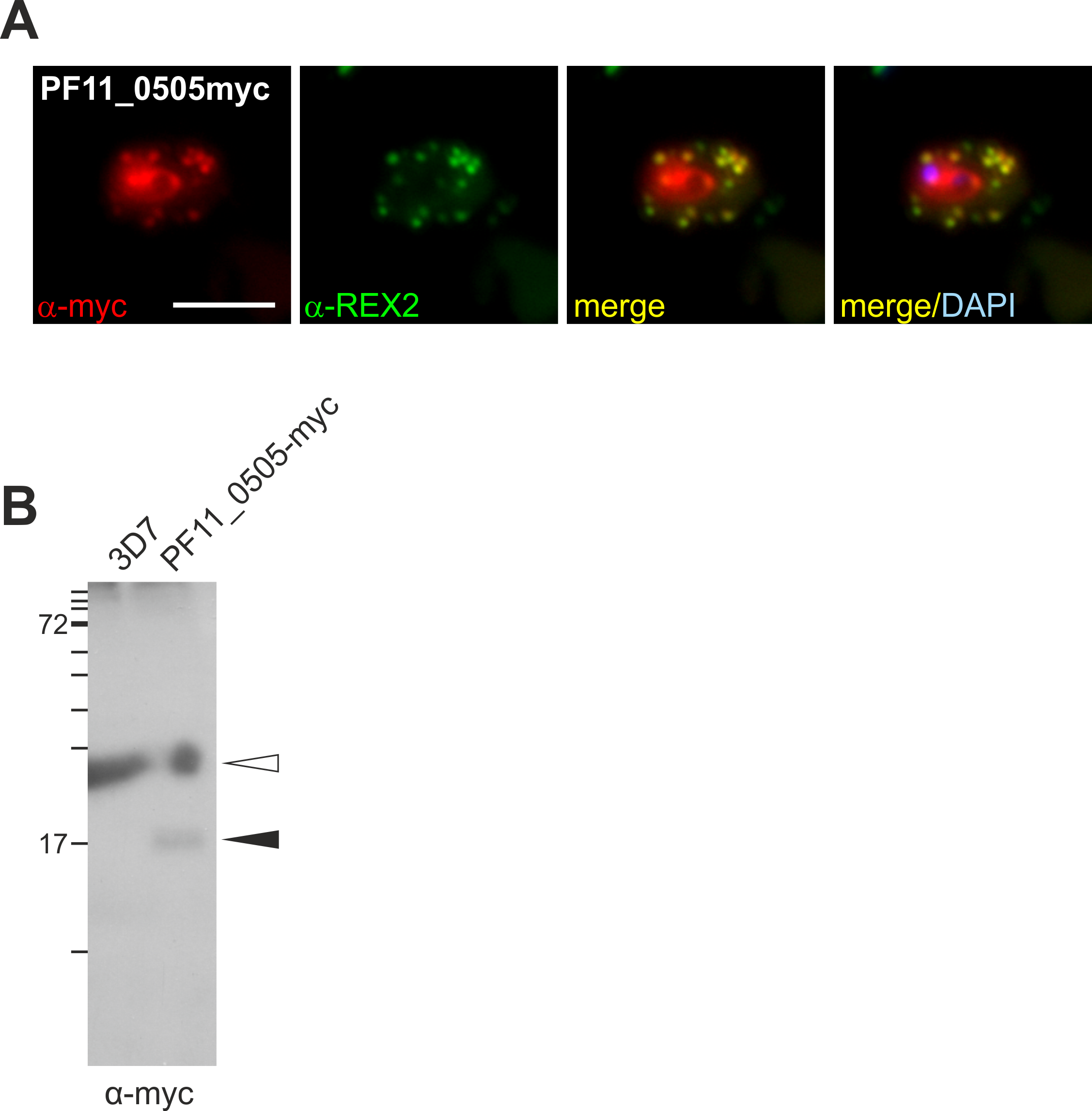

Supplement: Figure S3 — Export of a myc-tagged version of PF11_0505 to the Maurer's clefts. (A) IFA using anti-myc (red) and the Maurer's clefts marker REX2 (green) show co-location of PF11_0505myc and REX2 at the clefts. DAPI (blue) was used to stain nuclei. Size bars 5 µm. (B) Western blot probed with an anti-myc serum detects PF11_0505myc in the corresponding parasite line but not in 3D7 (filled arrowhead). The band apparent in both parasite extracts may be non-specific luminescence derived from left over hemoglobin (open arrowhead). The molecular weight standard is indicated as described in the legend to Figure S1. (TIF) [file ppat.1003546.s003.tif]

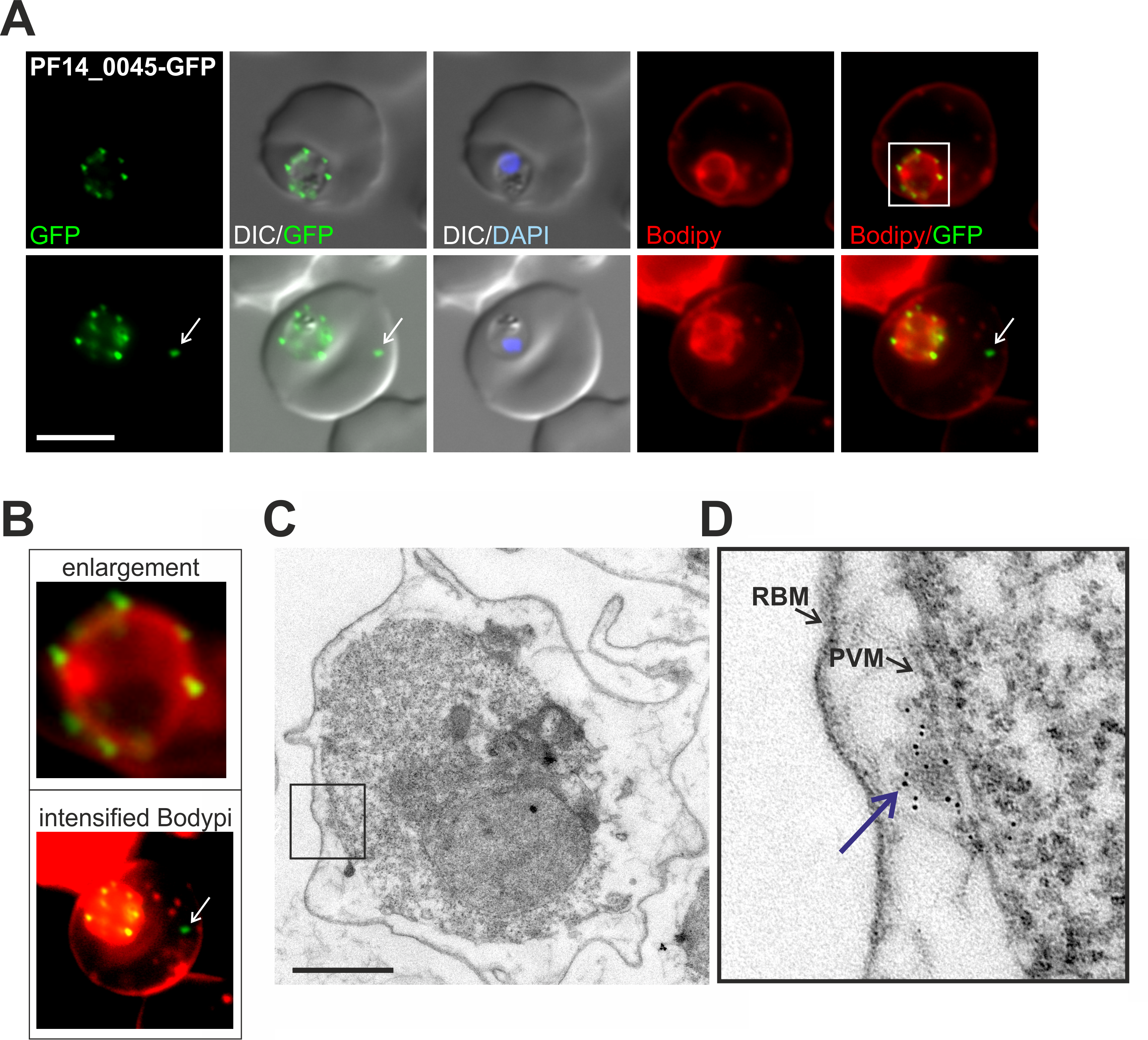

Supplement: Figure S4 — Localization of PF14_0045-GFP. (A) Fluorescence of PF14_0045-GFP (green) in Bodipy-TR-C5-ceramide (Bodipy, red) stained cells. Top panel cell showing GFP foci at the parasite periphery and bottom row a cell showing an additional focus in the host cell (white arrows). (B) Top, enlargement of the white frame from the top panel in (A) shows that the foci are parasite proximal but do not overlap and are located towards the host cell cytosol if compared to the Bodipy-TR-C5-ceramide staining. Bottom, the Bodipy-TR-C5-ceramide staining in the last image of the bottom row in (A) was intensified to demonstrate that the PF14_0045-GFP-derived focus in the host cell (white arrow) does not overlap with structures typically stained with Bodipy-TR-C5-ceramide such as Maurer's clefts. DAPI (blue) was used to stain nuclei. Size bars: 5 µm. (C) Pre-embedding immuno-EM (host cell cytosol released with Tetanolysin) using gold conjugated anti-GFP antibodies on PF14_0045-GFP expressing parasites. Size bar: 1 µm. The frame is enlarged in (D) and shows accumulation of gold in an electron dense area (blue arrow) that appears to be on the outside of the PVM. RBM (red blood cell membrane) and PVM are indicated. (TIF) [file ppat.1003546.s004.tif]

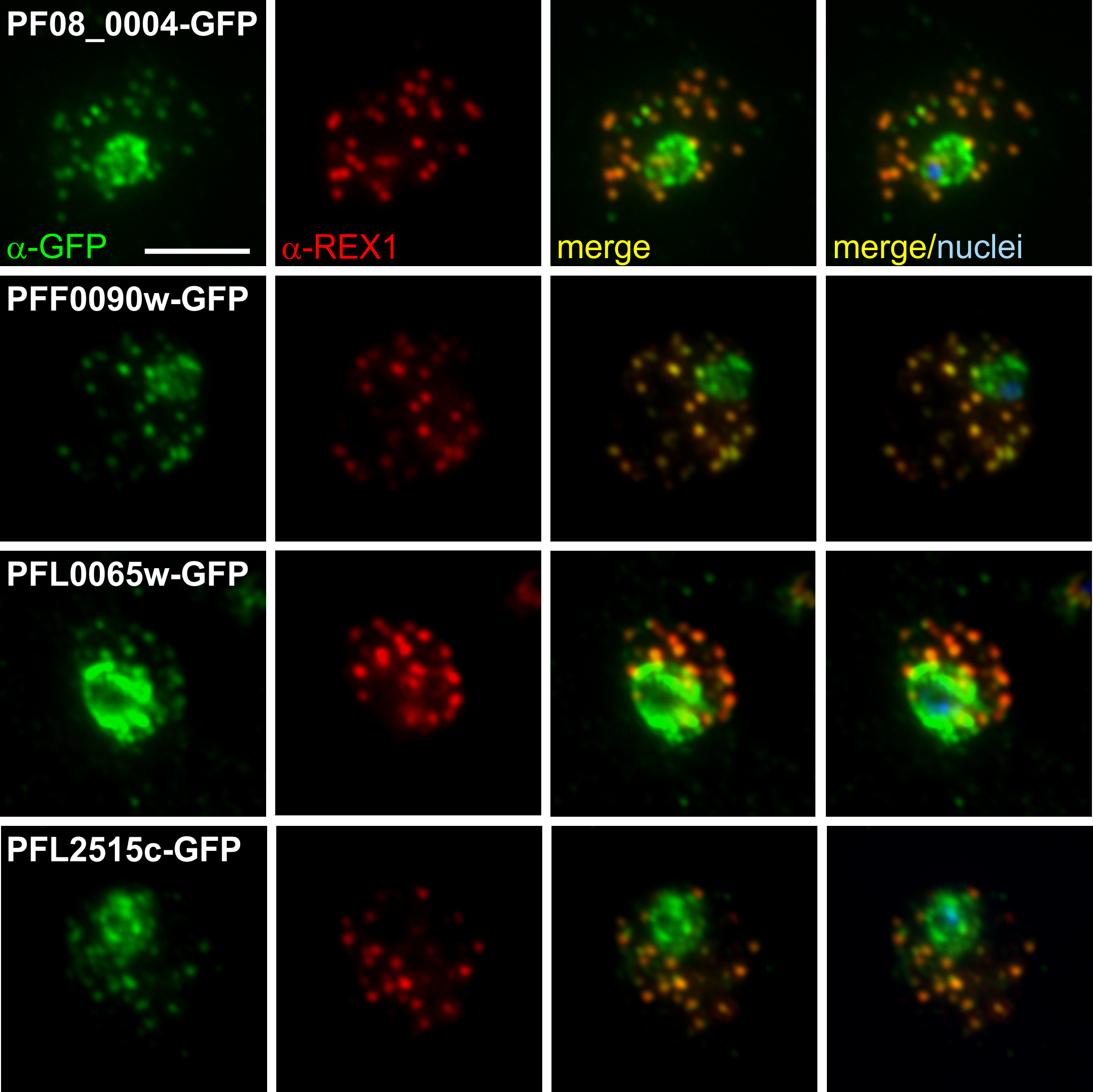

Supplement: Figure S5 — Co-localisation IFAs of GFP-tagged proteins from the genetic locus-based screen. The GFP-fusion proteins indicated on the right and detected with anti-GFP antibodies (green) co-locate with the Maurer's clefts Marker REX1 (red). DAPI (blue) was used to stain nuclei. Size bars: 5 µm. (TIF) [file ppat.1003546.s005.tif]

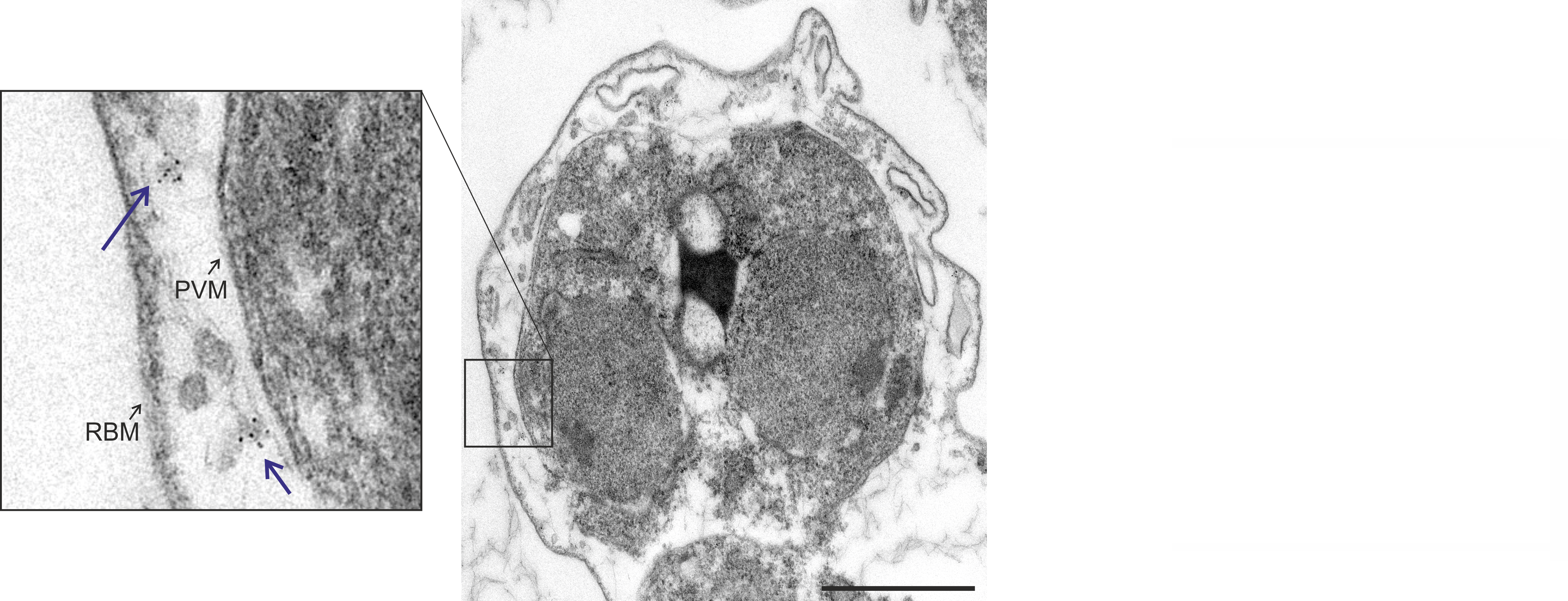

Supplement: Figure S6 — Immuno-EM analyis of PF13_0191-GFP expressing parasites. Pre-embedding immuno-EM (host cell cytosol released with Tetanolysin) using gold conjugated anti-GFP antibodies on PF13_0191-GFP expressing parasites. Size bars: 1 µm. The image to the left shows an enlargement of the region highlighted by a frame in the overview image. Accumulations of gold label in the host cell are indicated by blue arrows. RBM (red blood cell membrane) and PVM are indicated. (TIF) [file ppat.1003546.s006.tif]

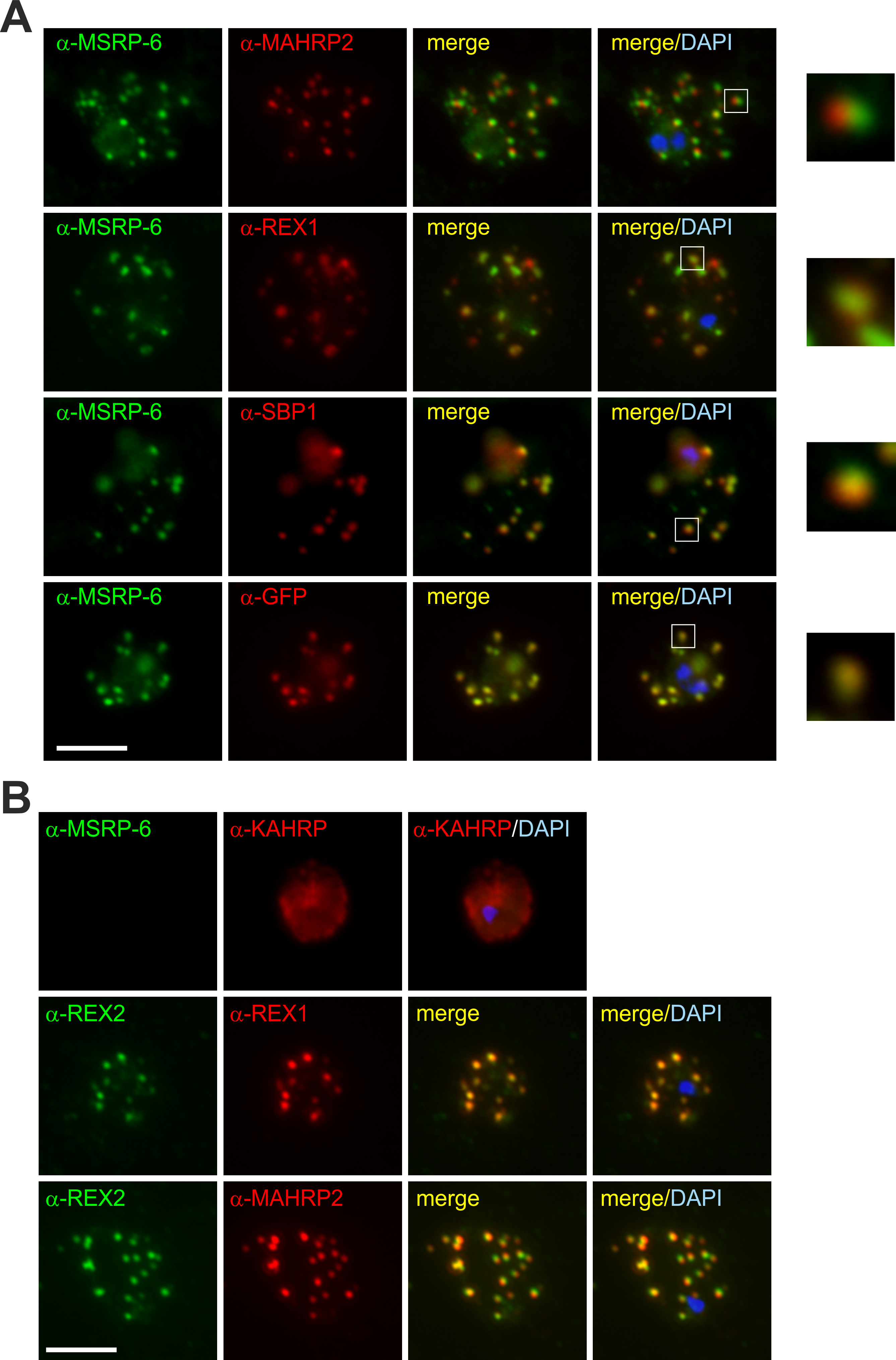

Supplement: Figure S7 — Co-localisation IFAs in 3D7 and MSRP6 knock out parasites. (A) IFAs with 3D7 parasites with the antisera indicated on the panels. The white frames highlight individual Maurer's clefts in the enlargements shown to the right. (B) IFAs with MSRP6 knock out parasites show no apparent change in the staining pattern typically obtained with the antisera indicated. DAPI (blue) was used to stain nuclei. Size bars 5 µm. (TIF) [file ppat.1003546.s007.tif]

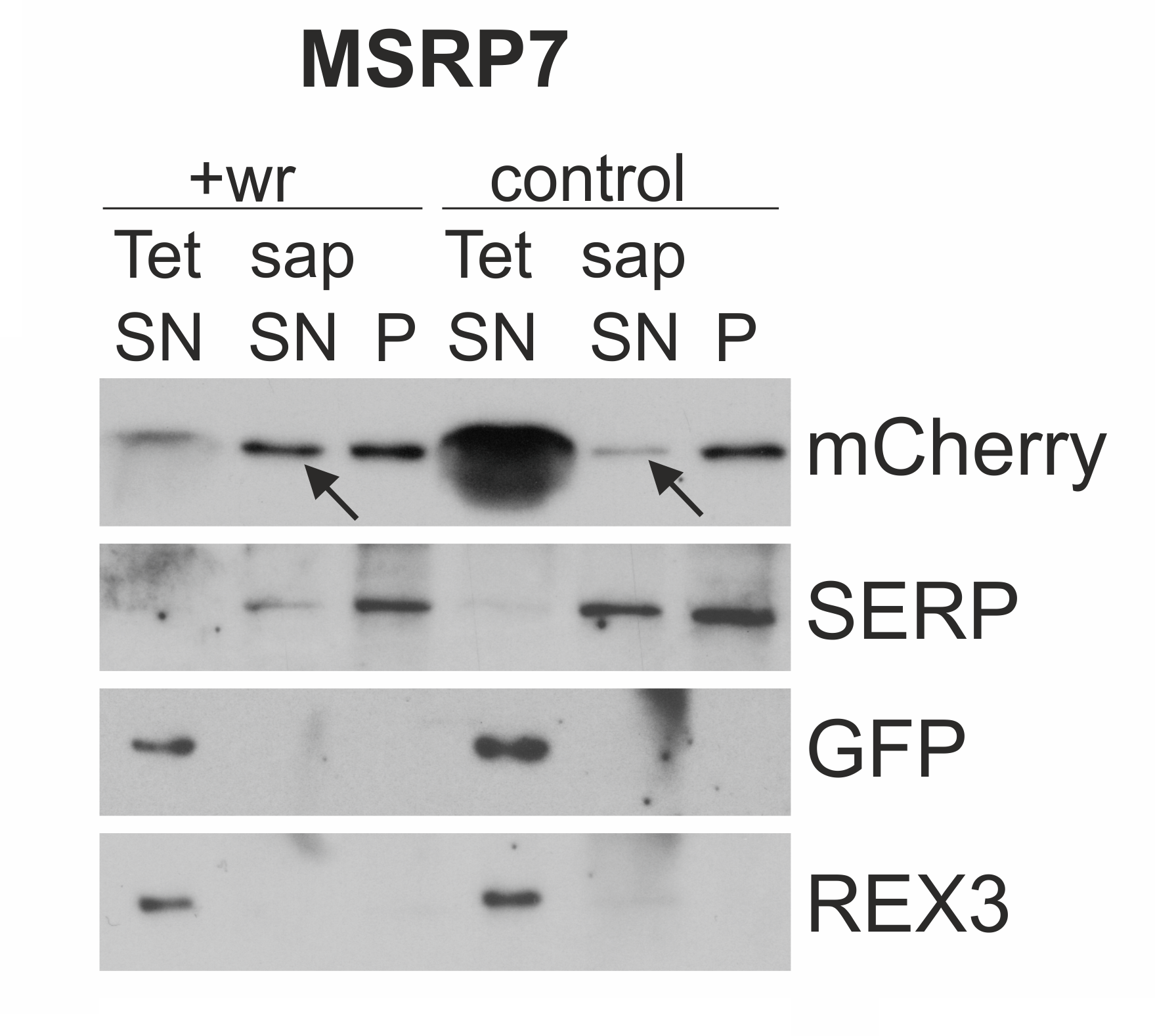

Supplement: Figure S8 — Export-blocked MSRP7-mDHFR-mCherry can be found soluble in the PV. Western blots of Percoll enriched double transgenic parasites expressing MSRP7-mDHFR-mCherry and MSRP7-GFP grown in the absence (control) or presence (+wr) of WR99210, treated sequentially with tetanolysin and saponin and separated into supernatant (SN) and pellet (P). REX3 is a parasite protein found in the host cell cytosol and was used as a control for host cell membrane lysis and release of this fraction. SERP is found soluble in the PV and was used to demonstrate the action of saponin. Note that not all of the SERP was released and hence the release of the export-blocked MSRP7-mDHFR-mCherry is equally incomplete. The presence of MSRP7-mDHFR-mCherry over MSRP7-GFP in the PV is highlighted by arrows. The distortion of the MSRP7-mDHFR-mCherry signal in the exported fraction is due to co-migration with BSA used in the tetanolysin lysis. The lower intensity of the mCherry, GFP and SERP signals in the extracts of parasites treated with wr may reflect either a slower growth cycle or differences in the stage composition of the parasite population after sample preparation. (TIF) [file ppat.1003546.s008.tif]
