# Supplementary material for: Identification of New PNEPs Indicates a Substantial Non-PEXEL Exportome and Underpins Common Features in Plasmodium falciparum Protein Export
Source: PLoS Pathog. 2013 Aug 8;9(8):e1003546. doi: 10.1371/journal.ppat.1003546 (PMC3738491; doi:10.1371/journal.ppat.1003546)
Supplement: Table S1 — 39 candidates with a similar transcription profile to known PNEPs. Yellow: selected candidates, green: ETRAMPs, blue: known PNEPs, grey: false-positives, orange: newly annotated PEXEL-proteins. (DOC) [file ppat.1003546.s009.doc]

**Table S1: 39 candidates with a similar transcription profile to known PNEPs**

| *Gene ID* | *Annotation in PlasmoDB5.4* | *Annotation in PlasmoDB7.2* |
| --- | --- | --- |
| PF07_0007 | hypothetical protein | conserved Plasmodium protein, unknown function |
| PF07_0008 | hypothetical protein | Plasmodium exported protein, unknown function |
| PF07_0011 | hypothetical protein, conserved | conserved Plasmodium protein, unknown function |
| PF08_0003 | tryptophan/threonine-rich antigen | tryptophan/threonine-rich antigen |
| PF08_0137 | hypothetical protein | Plasmodium exported protein (PHISTc), unknown function |
| PF10_0019 | early transcribed membrane protein | early transcribed membrane protein 10.1 |
| PF10_0024 | hypothetical protein | Plasmodium exported protein (hyp2), unknown function |
| PF10_0100 | hypothetical protein | conserved Plasmodium protein, unknown function[[1]](#endnote-2) |
| PF10_0350 | hypothetical protein | probable protein, unknown function[[2]](#endnote-3) |
| PF11_0039 | early transcribed membrane protein 11.1 | early transcribed membrane protein 11.1 |
| PF11_0040 | early transcribed membrane protein 11.2 | early transcribed membrane protein 11.2 |
| PF11_0175 | heat shock protein 101, putative | heat shock protein 101 |
| PF11_0505 | hypothetical protein | probable protein, unknown function |
| PF13_0194 | hypothetical protein | probable protein, unknown function |
| PF14_0016 | early transcribed membrane protein | early transcribed membrane protein 14.1 |
| PF14_0045 | hypothetical protein | conserved Plasmodium protein, unknown function |
| PF14_0076 | plasmepsin 1 precursor | plasmepsin I |
| PF14_0250 | hypothetical protein | lipase, putative |
| PF14_0344 | hypothetical protein | translocon component PTEX150 |
| PF14_0541 | V-type H+-translocating pyrophosphatase, putative | V-type H+-translocating pyrophosphatase, putative |
| PFB0120w | early transcribed membrane protein | early transcribed membrane protein 2 |
| PFB0485c | hypothetical protein | conserved Plasmodium protein, unknown function |
| PFB0900c | hypothetical protein | Plasmodium exported protein (PHISTc), unknown function |
| PFB0910w | hypothetical protein | Plasmodium exported protein, unknown function |
| PFB0915w | liver stage antigen 3 | liver stage antigen 3 |
| PFB0920w | hypothetical protein | DnaJ protein, putative |
| PFC0080c | hypothetical protein | Plasmodium exported protein (hyp1), unknown function |
| PFE0065w | skeleton-binding protein | skeleton-binding protein 1 |
| PFF1230c | hypothetical protein | conserved Plasmodium protein, unknown function |
| PFI1735c | hypothetical protein | ring-exported protein 1 |
| PFI1740c | hypothetical protein | ring-exported protein 2 |
| PFL0035c | octapeptide-repeat antigen, putative | acyl-CoA synthetase, PfACS7 |
| PFL1055c | hypothetical protein, conserved | conserved Plasmodium membrane protein, unknown function |
| PFL1410c | hypothetical protein | ABC transporter, (CT family) |
| PFL1945c | early transcribed membrane protein | early transcribed membrane protein 12 |
| MAL7P1.228 | heat shock 70kDa protein, (HSP70) | heat shock protein 70, putative |
| MAL7P1.27 | chloroquine resistance transporter, putative | chloroquine resistance transporter |
| MAL13P1.268 | hypothetical protein | conserved Plasmodium protein, unknown function |
| MAL13P1.413 | membrane associated histidine-rich protein, MAHRP-1 | membrane associated histidine-rich protein 1 |

1. Localized to ER as a GFP-fusion (S. Haase, unpublished) [↑](#endnote-ref-2)
2. Predicted GPI-anchored protein [↑](#endnote-ref-3)
