# Supplementary material for: Identification of New PNEPs Indicates a Substantial Non-PEXEL Exportome and Underpins Common Features in Plasmodium falciparum Protein Export
Source: PLoS Pathog. 2013 Aug 8;9(8):e1003546. doi: 10.1371/journal.ppat.1003546 (PMC3738491; doi:10.1371/journal.ppat.1003546)
Supplement: Table S2 — 397 candidates with a subtelomeric gene locus. (DOC) [file ppat.1003546.s010.doc]

**Table S2: 397 candidates with a subtelomeric gene locus**

| *Gene ID* | *Annotation in PlasmoDB7.0* |
| --- | --- |
| PFA0010c | rifin |
| PFA0020w | rifin |
| PFA0025c | null |
| PFA0070c | null |
| PFA0085c | null |
| PFA0105w | null |
| PFA0125c | erythrocyte binding antigen-181 |
| PFA0135w | Merozoite-associated tryptophan-rich antigen, putative |
| PFA0140c | conserved Plasmodium protein, unknown function |
| PFA0160c | nucleoside transporter, putative |
| PFA0175w | conserved Plasmodium protein, unknown function |
| PFA0180w | ATP-dependent RNA Helicase, putative |
| PFA0195w | conserved Plasmodium protein, unknown function |
| PFA0200w | Sporozoite stage TSP1 domain protein, S21 |
| PFA0205w | conserved Plasmodium membrane protein, unknown function |
| PFA0210c | conserved Plasmodium protein, unknown function |
| PFA0580c | TatD-like deoxyribonuclease, putative |
| PFA0590w | ABC transporter, (CT family), putative |
| PFA0625w | Surface-associated interspersed gene 1.1 (SURFIN 1.1) |
| PFA0645c | hypothetical protein |
| PFA0690w | null |
| PFA0695c | null |
| PFA0705c | null |
| PFA0720w | hypothetical protein |
| PFA0725w | Surface-associated interspersed protein 1.3 (SURFIN 1.3) |
| PFB0020c | erythrocyte membrane protein 1 (PfEMP1), exon2 |
| PFB0030c | rifin |
| PFB0045c | erythrocyte membrane protein 1 (PfEMP1), truncated |
| PFB0050c | stevor pseudogene |
| PFB0110w | Plasmodium exported protein (hyp11), unknown function |
| PFB0120w | early transcribed membrane protein 2, ETRAMP2 |
| PFB0140w | zinc finger protein, putative |
| PFB0170w | conserved Plasmodium protein, unknown function |
| PFB0180w | 5'-3' exonuclease, N-terminal resolvase-like domain, putative |
| PFB0190c | conserved Plasmodium protein, unknown function |
| PFB0194w | conserved Plasmodium protein, unknown function |
| PFB0855c | apicoplast RNA methyltransferase precursor, putative |
| PFB0877c | probable protein, unknown function |
| PFB0886c | conserved Plasmodium protein, unknown function |
| PFB0888w-a | ribosome associated membrane protein RAMP4, putative |
| PFB0888w-b | ribosome associated membrane protein RAMP4, putative |
| PFB0890c | pseudouridine synthase, putative |
| PFB0900c | Plasmodium exported protein (PHISTc), unknown function |
| PFB0910w | Plasmodium exported protein, unknown function |
| PFB0915w | liver stage antigen 3 |
| PFB0925w | DNAJ protein, putative |
| PFB0926c | Plasmodium exported protein (hyp2), unknown function |
| PFB0935w | cytoadherence linked asexual protein 2 |
| PFB0950w | conserved Plasmodium falciparum protein family |
| PFB0960c | Pfmc-2TM family pseudogene |
| PFB0976w | hypothetical protein |
| PFB0980w | conserved Plasmodium falciparum protein family |
| PFB0995w | null |
| PFB1000w | rifin |
| PFB1010w | rifin |
| PFB1040w | rifin |
| PFB1045w | erythrocyte membrane protein 1 (PfEMP1), truncated |
| PFC0010c | rifin |
| PFC0015c | null |
| PFC0050c | acyl-CoA synthetase, PfACS2 |
| PFC0055w | Plasmodium exported protein (hyp13), unknown function |
| PFC0095c | conserved Plasmodium protein, unknown function |
| PFC0110w | Cytoadherence linked asexual protein 3.2 |
| PFC0115c | null |
| PFC0120w | Cytoadherence linked asexual protein 3.1 |
| PFC0125w | ABC transporter, (TAP family), putative |
| PFC0160w | binding protein, putative |
| PFC0176c | conserved Plasmodium protein, unknown function |
| PFC0912w | signal peptidase, putative |
| PFC0925w | endonuclease, putative |
| PFC0935c | N-acetylglucosamine-1-phosphate transferase, putative |
| PFC0940c | conserved Plasmodium protein, unknown function |
| PFC0945w | protein kinase, putative |
| PFC0970w | conserved Plasmodium membrane protein, unknown function |
| PFC0995c | acyl CoA:diacylglycerol acyltransferase, putative |
| PFC1000w | regulator of initiation factor 2 (eIF2) |
| PFC1010w | conserved Plasmodium protein, unknown function |
| PFC1011c | conserved Plasmodium membrane protein, unknown function |
| PFC1015c | conserved Plasmodium protein, unknown function |
| PFC1030w | conserved Plasmodium protein, unknown function |
| PFC1035w | conserved Plasmodium protein, unknown function |
| PFC1045c | conserved Plasmodium protein, unknown function |
| PFC1070c | null |
| PFC1075w | hypothetical protein |
| PFC1110w | null |
| PFD0015c | rifin |
| PFD0025w | rifin |
| PFD0028w | null |
| PFD0030c | rifin |
| PFD0050w | rifin |
| PFD0065w | null |
| PFD0075w | conserved Plasmodium falciparum protein family |
| PFD0085c | acyl-CoA synthetase, PfACS6 |
| PFD0110w | reticulocyte-binding protein homologue 1 |
| PFD0120w | null |
| PFD0134c | null |
| PFD0140w | null |
| PFD0144c | probable protein, unknown function |
| PFD0155c | conserved Plasmodium protein, unknown function |
| PFD0160w | conserved Plasmodium protein, unknown function |
| PFD1037w | conserved Plasmodium protein, unknown function |
| PFD1075w | serpentine receptor, putative |
| PFD1100c | conserved Plasmodium protein, unknown function |
| PFD1105w | asparagine-rich protein |
| PFD1110w | conserved Plasmodium membrane protein, unknown function |
| PFD1120c | early transcribed membrane protein 4, ETRAMP4 |
| PFD1130w | conserved Plasmodium protein, unknown function |
| PFD1135c | probable protein, unknown function |
| PFD1145c | reticulocyte-binding protein homologue 5 |
| PFD1150c | reticulocyte binding protein homolog 4, Rh4 |
| PFD1155w | erythrocyte binding antigen-165 |
| PFD1195c | conserved Plasmodium protein, unknown function, pseudogene |
| PFD1205w | Plasmodium exported protein (hyp15), unknown function |
| PFD1225w | null |
| PFD1230c | rifin |
| PFD1240w | rifin |
| PFE0010c | null |
| PFE0015c | null |
| PFE0025c | rifin |
| PFE0030c | null |
| PFE0065w | skeleton-binding protein 1 |
| PFE0075c | rhoptry-associated protein 3, RAP3 |
| PFE0080c | rhoptry-associated protein 2, RAP2 |
| PFE0110w | conserved Plasmodium membrane protein, unknown function |
| PFE0120c | Merozoite Surface Protein 8, MSP8 |
| PFE0125w | conserved Plasmodium protein, unknown function |
| PFE0145w | organelle ribosomal protein L28 precursor, putative |
| PFE0150c | 4-diphosphocytidyl-2c-methyl-D-erythritol kinase (CMK), putative |
| PFE0190c | conserved Plasmodium protein, unknown function |
| PFE0195w | cation transporting P-ATPase |
| PFE0205w | ATP-dependent helicase, putative |
| PFE0220w | conserved Plasmodium protein, unknown function |
| PFE1405c | eukaryotic translation initiation factor 3, subunit 6, putative |
| PFE1415w | cell cycle regulator with zn-finger domain, putative |
| PFE1440c | conserved Plasmodium protein, unknown function |
| PFE1445c | conserved Plasmodium protein, unknown function |
| PFE1450c | conserved Plasmodium protein, unknown function |
| PFE1455w | sugar transporter, putative |
| PFE1460w | apicoplast TIC22 precursor, putative |
| PFE1490c | RING zinc finger protein, putative |
| PFE1505w | SNARE protein, putative |
| PFE1510c | triose phosphate transporter |
| PFE1515w | conserved Plasmodium membrane protein, unknown function |
| PFE1525w | conserved Plasmodium membrane protein, unknown function |
| PFE1540w | CPW-WPC family protein |
| PFE1550w | conserved Plasmodium protein, unknown function |
| PFE1590w | early transcribed membrane protein 5, ETRAMP5 |
| PFE1595c | Plasmodium exported protein (PHISTc), unknown function |
| PFE1635w | null |
| PFF0015c | rifin |
| PFF0030c | erythrocyte membrane protein 1(PfEMP1) pseudogene |
| PFF0035c | rifin |
| PFF0045c | RIF pseudogene |
| PFF0065c | null |
| PFF0070w | null |
| PFF0085w | Plasmodium exported protein (PHISTa), unknown function, pseudogene |
| PFF0090w | conserved Plasmodium protein, unknown function |
| PFF0110w | conserved Plasmodium protein, unknown function |
| PFF0115c | elongation factor G, putative |
| PFF0155w | Bcs1 protein, putative |
| PFF0160c | dihydroorotate dehydrogenase, mitochondrial precursor |
| PFF0170w | calcium antiporter, putative |
| PFF0175c | conserved Plasmodium protein, unknown function |
| PFF0185c | conserved Plasmodium protein, unknown function |
| PFF0215w | conserved Plasmodium protein, unknown function |
| PFF1455c | CPW-WPC family protein |
| PFF1465w | conserved Plasmodium protein, unknown function |
| PFF1475c | conserved Plasmodium protein, unknown function |
| PFF1515c | null |
| PFF1520w | RESA-like protein |
| PFF1540w | null |
| PFF1565c | rifin |
| PFF1585w | null |
| PFF1590w | rifin |
| MAL7P1.216 | rifin |
| MAL7P1.217 | rifin |
| MAL7P1.219 | rifin |
| MAL7P1.223 | stevor, putative |
| MAL7P1.226 | null |
| MAL7P1.230 | hypothetical protein, pseudogene |
| MAL7P1.229 | Cytoadherence linked asexual protein |
| MAL7P1.200 | rifin |
| PF07_0002 | RESA-like protein |
| PF07_0004 | Plasmodium exported protein, unknown function |
| PF07_0007 | conserved Plasmodium protein, unknown function |
| PF07_0008 | Plasmodium exported protein, unknown function |
| MAL7P1.10 | centrin, putative |
| PF07_0010 | conserved Plasmodium protein, unknown function |
| PF07_0011 | conserved Plasmodium protein, unknown function |
| MAL7P1.12 | erythrocyte membrane-associated antigen |
| MAL7P1.159 | 1-cys peroxiredoxin |
| MAL7P1.163 | conserved Plasmodium protein, unknown function |
| MAL7P1.170 | Plasmodium exported protein, unknown function |
| MAL7P1.176 | erythrocyte binding antigen 175 |
| PF07_0129 | acyl-coA synthetase, PfACS5 |
| MAL7P1.178 | alpha/beta hydrolase, putative |
| MAL7P1.179 | null |
| MAL7P1.183 | null |
| MAL7P1.186 | VAR-like erythrocyte membrane protein 1 |
| PF07_0138 | rifin |
| PF08_0139 | rifin |
| PF08_0137 | Plasmodium exported protein (PHISTc), unknown function |
| PF08_0136b | von Willebrand factor A-domain-related protein, putative |
| PF08_0134 | conserved Plasmodium protein, unknown function |
| MAL8P1.156 | mannose-6-phosphate isomerase, putative |
| MAL8P1.152 | conserved Plasmodium protein, unknown function |
| MAL8P1.151 | inositol phosphatase, putative |
| PF08_0011 | leucine - tRNA ligase |
| MAL8P1.14 | mitochondrial inner membrane translocase, putative |
| MAL8P1.13 | folate/biopterin transporter, putative |
| PF08_0008 | conserved Plasmodium protein, unknown function |
| MAL8P1.11 | conserved Plasmodium membrane protein, unknown function |
| PF08_0006 | prohibitin, putative |
| MAL8P1.7 | conserved Plasmodium protein, unknown function |
| MAL8P1.6 | early transcribed membrane protein 8, ETRAMP 8 |
| PF08_0005 | conserved Plasmodium protein, unknown function |
| PF08_0004 | conserved Plasmodium protein, unknown function |
| PF08_0003 | tryptophan/threonine-rich antigen |
| MAL8P1.3 | Plasmodium exported protein (hyp9), unknown function |
| PF08_0002 | surface-associated interspersed gene 8.2 (SURFIN8.2) |
| MAL8P1.2 | Plasmodium exported protein (PHISTb), unknown function |
| MAL8P1.1 | surface-associated interspersed gene 8.1, (SURFIN8.1) |
| MAL8P1.204 | DNAJ protein, putative |
| MAL8P1.208 | rifin |
| MAL8P1.210 | hypothetical protein conserved in P. falciparum |
| MAL8P1.211 | hypothetical protein conserved in P. falciparum |
| MAL8P1.214 | stevor, pseudogene |
| MAL8P1.217 | stevor |
| MAL8P1.218 | rifin |
| PFI0040c | null |
| PFI0065w | rifin |
| PFI0090c | probable protein, unknown function |
| PFI0095c | Serine/Threonine protein kinase, FIKK family |
| PFI0105c | Serine/Threonine protein kinase, FIKK family |
| PFI0125c | Serine/Threonine protein kinase, FIKK family |
| PFI0135c | serine repeat antigen 9 (SERA-9) |
| PFI0140w | conserved Plasmodium protein, unknown function |
| PFI0145w | conserved protein, unknown function |
| PFI0150c | retrieval receptor for endoplasmic reticulum membrane proteins, putative |
| PFI0160w | conserved Plasmodium membrane protein, unknown function |
| PFI0185w | conserved Plasmodium protein, unknown function |
| PFI0205w | conserved Plasmodium protein, unknown function |
| PFI0210c | cysteine repeat modular protein, putative |
| PFI1645c | histidyl-tRNA synthetase, putative |
| PFI1660w | conserved Plasmodium protein, unknown function |
| PFI1696c | conserved protein, unknown function |
| PFI1705w | phosphatidylinositol N-acetylglucosaminyltransferase subunit P, putative |
| PFI1730w | cytoadherence linked asexual protein 9(CLAG9) |
| PFI1735c | ring-exported protein 1 |
| PFI1740c-a | null |
| PFI1740c-b | ring-exported protein 2 |
| PFI1745c | early transcribed membrane protein (ETRAMP) |
| PFI1750c | Plasmodium exported protein (hyp11), unknown function |
| PFI1790w | Plasmodium exported protein (PHISTb), unknown function |
| PFI1815c | rifin |
| PFI1825w | rifin |
| PF10_0002 | rifin |
| PF10_0005 | rifin |
| PF10_0007 | Plasmodium exported protein, unknown function, pseudogene |
| PF10_0009 | stevor, pseudogene |
| PF10_0012 | erythrocyte membrane protein 1 (PfEMP1), truncated |
| PF10_0019 | early transcribed membrane protein 10.1, etramp 10.1 |
| PF10_0024 | Plasmodium exported protein (hyp2), unknown function |
| PF10_0026 | Tryptophan-rich antigen 3, putative |
| PF10_0027 | conserved Plasmodium protein, unknown function |
| PF10_0030 | conserved Plasmodium protein, unknown function |
| PF10_0032 | DNAJ protein, putative |
| PF10_0033 | conserved Plasmodium protein, unknown function |
| PF10_0034 | conserved Plasmodium protein, unknown function |
| PF10_0034a | conserved Plasmodium protein, unknown function |
| PF10_0035 | conserved Plasmodium protein, unknown function |
| PF10_0037 | conserved Plasmodium protein, unknown function |
| PF10_0045 | conserved Plasmodium membrane protein, unknown function |
| PF10_0371 | conserved Plasmodium protein, unknown function |
| PF10_0415 | conserved Plasmodium protein, unknown function |
| PF10_0372 | Antigen UB05 |
| PF10_0374 | Pf11-1 protein |
| PF10_0384 | hypothetical protein, pseudogene |
| PF10_0388 | hypothetical protein, pseudogene |
| PF10_0399 | rifin |
| PF10_0403 | rifin |
| PF10_0405 | rifin |
| PF11_0009 | rifin |
| PF11_0012 | null |
| PF11_0013 | stevor, putative, degenerate |
| PF11_0015 | hypothetical protein, pseudogene |
| PF11_0529 | rifin |
| PF11_0021 | rifin |
| PF11_0022 | rifin, pseudogene |
| PF11_0026 | hypothetical protein |
| PF11_0039 | early transcribed membrane protein 11.1, etramp11.1 |
| PF11_0040 | early transcribed membrane protein 11.2, etramp11.2 |
| PF11_0042 | conserved Plasmodium protein, unknown function |
| PF11_0044 | iron-sulfur assembly protein, sufD, putative |
| PF11_0045 | CPW-WPC family protein |
| PF11_0049 | NOT family protein, putative |
| PF11_0052 | Qa-SNARE protein, putative |
| PF11_0055 | conserved protein, unknown function |
| PF11_0479a | conserved Plasmodium membrane protein, unknown function |
| PF11_0483a | conserved Plasmodium protein, unknown function |
| PF11_0486 | MAEBL, putative |
| PF11_0487 | conserved Plasmodium protein, unknown function |
| PF11_0504 | Plasmodium exported protein (hyp11), unknown function |
| PF11_0505 | probable protein, unknown function |
| PF11_0518 | null |
| PF11_0520 | rifin |
| PFL0035c | acyl-CoA synthetase, PfACS7 |
| PFL0065w | conserved Plasmodium protein, unknown function |
| PFL0080c | serine/threonine-protein kinase, Pfnek-3 |
| PFL0085c | conserved Plasmodium membrane protein, unknown function |
| PFL0110c | mitochondrial phosphate carrier protein |
| PFL0125c | conserved Plasmodium protein, unknown function |
| PFL0170w | transporter, putative |
| PFL2455w | conserved Plasmodium protein, unknown function |
| PFL2470c | conserved Plasmodium protein, unknown function |
| PFL2485c | tryptophanyl-tRNA synthetase, putative |
| PFL2505c | rhoptry neck protein 3, putative |
| PFL2510w | chitinase |
| PFL2515c | conserved Plasmodium protein, unknown function |
| PFL2520w | reticulocyte-binding protein 3 homologue |
| PFL2525c | Plasmodium exported protein, unknown function |
| PFL2570w | acyl-CoA synthetase, PfACS3 |
| PFL2575c | Plasmodium exported protein, unknown function |
| PFL2595w | Plasmodium exported protein (PHISTa), unknown function |
| PFL2630w | rifin |
| PFL2640c | rifin |
| PFL2645c | rifin |
| PFL2660w | rifin |
| MAL13P1.6 | erythrocyte membrane protein 1-like |
| MAL13P1.8 | RIF pseudogene |
| MAL13P1.11 | Plasmodium exported protein (PHISTa), unknown function |
| MAL13P1.59 | Plasmodium exported protein (PHISTa), unknown function |
| MAL13P1.60 | erythrocyte binding antigen-140 |
| PF13_0075 | surface-associated interspersed gene 13.1 (SURFIN13.1) |
| MAL13P1.62 | Plasmodium exported protein, unknown function |
| PF13_0012 | early transcribed membrane protein 13, ETRAMP13 |
| PF13_0012a | Plasmodium exported protein, unknown function |
| MAL13P1.12 | conserved Plasmodium membrane protein, unknown function |
| MAL13P1.15 | conserved Plasmodium protein, unknown function |
| MAL13P1.16 | SNARE protein, putative |
| PF13_0019 | sodium/hydrogen exchanger, Na+, H+ antiporter |
| MAL13P1.19 | peptidase, putative |
| PF13_0354 | alanine--tRNA ligase, putative |
| PF13_0355 | conserved Plasmodium protein, unknown function |
| MAL13P1.342 | conserved Plasmodium protein, unknown function |
| PF13_0359 | mitochondrial carrier protein, putative |
| MAL13P1.348 | conserved Plasmodium membrane protein, unknown function |
| MAL13P1.351 | conserved Plasmodium protein, unknown function |
| PF13_0361 | conserved Plasmodium protein, unknown function |
| MAL13P1.413 | membrane associated histidine-rich protein, MAHRP-1 |
| MAL13P1.415 | null |
| MAL13P1.425 | hypothetical protein |
| MAL13P1.461 | probable protein, unknown function |
| MAL13P1.465 | Plasmodium exported protein, unknown function |
| MAL13P1.475 | Plasmodium exported protein (PHISTb), unknown function |
| MAL13P1.485 | acyl-CoA synthetase, PfACS4 |
| MAL13P1.495 | rifin |
| MAL13P1.510 | null |
| PF14_0016 | early transcribed membrane protein 14.1, etramp14.1 |
| PF14_0022 | exopolyphosphatase, putative |
| PF14_0023 | conserved Plasmodium protein, unknown function |
| PF14_0024 | conserved Plasmodium protein, unknown function |
| PF14_0029a | conserved Plasmodium protein, unknown function |
| PF14_0031a | conserved Plasmodium protein, unknown function |
| PF14_0031b | conserved Plasmodium protein, unknown function |
| PF14_0033 | Sel1 protein |
| PF14_0034 | translocation associated membrane protein, putative |
| PF14_0035 | conserved Plasmodium protein, unknown function |
| PF14_0040 | Secreted ookinete adhesive protein |
| PF14_0788 | adenylyl cyclase 1, putative |
| PF14_0044 | conserved Plasmodium protein, unknown function |
| PF14_0045 | conserved Plasmodium protein, unknown function |
| PF14_0046 | conserved Plasmodium protein, unknown function |
| PF14_0048 | conserved Plasmodium protein, unknown function |
| PF14_0051 | DNA mismatch repair protein, putative |
| PF14_0054 | conserved protein, unknown function |
| PF14_0722 | cysteine repeat modular protein 4, putative |
| PF14_0723 | LCCL domain-containing protein CCP1 |
| PF14_0726 | conserved Plasmodium protein, unknown function |
| PF14_0728 | conserved Plasmodium protein, unknown function |
| PF14_0729 | early transcribed membrane protein 14.2, etramp14.2 |
| PF14_0733 | Serine/Threonine protein kinase, FIKK family |
| PF14_0734 | Serine/Threonine protein kinase, FIKK family |
| PF14_0735 | probable protein, unknown function |
| PF14_0741 | hypothetical protein |
| PF14_0742 | Plasmodium exported protein (hyp6), unknown function |
| PF14_0745 | probable protein, unknown function |
| PF14_0747 | surface -associated intersprsed gene 14.1 (SURFIN 14.1) |
| PF14_0748 | Plasmodium exported protein (PHISTa), unknown function |
| PF14_0751 | acyl-CoA synthetase, PfACS1b |
| PF14_0754 | hypothetical protein |
| PF14_0756 | probable protein, unknown function |
| PF14_0759 | conserved Plasmodium protein, unknown function, pseudogene |
| PF14_0761 | acyl-CoA synthetase |
| PF14_0762 | Plasmodium exported protein, unknown function |
| PF14_0764 | Plasmodium exported protein (PHISTa), unknown function |
| PF14_0769 | rifin |
