# Supplementary material for: Identification of New PNEPs Indicates a Substantial Non-PEXEL Exportome and Underpins Common Features in Plasmodium falciparum Protein Export
Source: PLoS Pathog. 2013 Aug 8;9(8):e1003546. doi: 10.1371/journal.ppat.1003546 (PMC3738491; doi:10.1371/journal.ppat.1003546)
Supplement: Table S3 — Candidates selected for GFP-tagging. (DOC) [file ppat.1003546.s011.doc]

**Table S3: Candidates selected for GFP-tagging**

| *Gene ID* | *Annotation* | *Gene structure* | *Coding sequence [bp]* | *Hydrophobic region(s)* | *Molecular weight [kDa]* |
| --- | --- | --- | --- | --- | --- |
| PF07_0007 | hypothetical protein | 2 Exons | 372 | 1 TMD | 14 |
| PF07_0008 | hypothetical protein | 2 Exons | 762 | 1 TMD | 28 |
| PF07_0011 | hypothetical protein, conserved | 1 Exon | 543 | 1 TMD | 21 |
| PF08_0003 | tryptophan/threonine-rich antigen | 2 Exons | 2028 | 1 TMD | 80 |
| PF11_0175 | heat shock protein 101, putative | 4 Exons | 2721 | SP | 103 |
| PF11_0505 | hypothetical protein | 1 Exon | 270 | 2 TMDs | 10 |
| PF13_0194 | hypothetical protein | 1 Exon | 609 | SP | 23 |
| PF14_0045 | hypothetical protein | 1 Exon | 2895 | SP | 114 |
| PF14_0250 | hypothetical protein | 1 Exon | 3963 | SP | 156 |
| PFA0420w | hypothetical protein | 1 Exon | 540 | - | 20 |
| PFB0485c | hypothetical protein | 1 Exon | 1248 | SP, 2 TMDs | 50 |
| PFF1230c | hypothetical protein | 1 Exon | 1239 | SP | 49 |
| PFL1055c | hypothetical protein, conserved | 1 Exon | 2580 | SP, 4 TMDs | 101 |
| PF13_0191 | hypothetical protein, conserved | 1 Exon | 1380 | SP | 54 |
| PF13_0192 | hypothetical protein, conserved | 1 Exon | 1782 | SP | 71 |
| PFL2515c | protein with unknown function | 2 Exons | 258 | 1 TMD | 9 |
| PF07_0010 | protein with unknown function | 1 Exon | 5532 | 1 TMD | 220 |
| PF08_0004 | protein with unknown function | 2 Exons | 414 | SP, 1 TMD | 16 |
| PF08_0005 | protein with unknown function | 1 Exon | 1167 | SP | 46 |
| PF14_0024 | protein with unknown function | 1 Exon | 567 | 1 TMD | 22 |
| PF14_0044 | protein with unknown function | 1 Exon | 873 | SP | 33 |
| PF14_0046 | protein with unknown function | 1 Exon | 894 | SP | 35 |
| PFC1035w | protein with unknown function | 1 Exon | 1326 | 1 TMD | 52 |
| PFF0090w | protein with unknown function | 2 Exons | 387 | 1 TMD | 15 |
| PFL0065w | protein with unknown function | 1 Exon | 321 | SP, 1 TMD | 12 |
