# Supplementary material for: Identification of New PNEPs Indicates a Substantial Non-PEXEL Exportome and Underpins Common Features in Plasmodium falciparum Protein Export
Source: PLoS Pathog. 2013 Aug 8;9(8):e1003546. doi: 10.1371/journal.ppat.1003546 (PMC3738491; doi:10.1371/journal.ppat.1003546)
Supplement: Table S4 — Primers used in this study. (DOC) [file ppat.1003546.s012.doc]

**Table S4: Primers used in this study^*^**

| *Primer name* | *Primer sequence (5´- 3´)* |
| --- | --- |
| PF07_0007-KpnI-fw | CAGCGGTACCGAAAATGAGTCAACCACAAAAACAAC |
| PF07_0007-AvrII-rv | CAGCCCTAGGTTTTGCGTTCTGTAAACTGGCTTG |
| PF07_0008-KpnI-fw | CAGCGGTACCAAAGATGGCTTATCCTCTTTTAGAAG |
| PF07_0008-AvrII-rv | CAGCCCTAGGTACATGAGCTTCATTAGTGTTTAAAC |
| PF07_0011-KpnI-fw | CAGCGGTACCTAATATGAATAATAATAATTACATATTTG |
| PF07_0011-AvrII-rv | CAGCCCTAGGAAATTTAATAATTGGATAGTGATAATTAATAC |
| PF08_0003-KpnI-fw | CAGCGGTACCTAAAATGAATTTAGAGCAGTTTA |
| PF08_0003-AvrII-rv | CAGCCCTAGGAACATTAATTTCATTTCCGTCATC |
| PF11_0175-KpnI-fw | CAGCGGTACCTAAAAAATGACAAGAAGATATTTAAAG |
| PF11_0175-AvrII-rv | CAGCCCTAGGGGTCTTAGATAAGTTTATAACCAAG |
| PF11_0505-KpnI-fw | CAGCGGTACCAGAAATGGAAGCTGAGAAAAAGG |
| PF11_0505-AvrII-rv | CAGCCCTAGGTTTAAAGAACGTTACATAAAATGC |
| PF11_0505-myc-XhoI-rv | TGGCCTCGAGTTATAAATCTTCTTCGCTTATGAGTTTTTGTTCCCTAGGTTTAAAGAACGTTACATAAAATGCTG |
| PF13_0194-KpnI-fw | CAGCGGTACCTACAATGAAAAGAACAAGACATATTAC |
| PF13_0194-AvrII-rv | CAGCCCTAGGTTGTAAGGTATCTAAATGAGAAAG |
| PF14_0045-KpnI-fw2 | CAGCGGTACCTATATATGTATATATATATATATATACAAGTACCAATGAAGATTTTATTTAATAATACTTTTGAACTC |
| PF14_0045-AvrII-rv1506 | CAGCCCTAGGGGCTTCATGTATATCTTCTCTATCAC |
| PF14_0250-KpnI-fw | CAGCGGTACCGAAAATGTTACCATGCGTGATAATTTCC |
| PF14_0250-AvrII-rv | CAGCCCTAGGATCACTGTTGTAGTTATTGTATTTCTCGG |
| PFA0420w-KpnI-fw | CAGCGGTACCAAAAATGGCAAAAAGAAAATATATGTC |
| PFA0420w-AvrII-rv813 | TTTTCTTTTTTGTAAATCCTAGGTTCATTTGCTGG |
| PFB0485c-KpnI-fw | CAGCGGTACCAAAAATGAAGATAAGTTTTTTATTTAAGTTAGTTTG |
| PFB0485c-AvrII-rv | CAGCCCTAGGATAAAAAGGGAAAGAATAAATATCTTTTAAG |
| PFF1230c-KpnI-fw | CAGCGGTACCCAAATATGAAATTCTTCGTACTGTTTTTG |
| PFF1230c-AvrII-rv | CAGCCCTAGGTAACTCCACATTTTTTTTTGGGAAATATATTAG |
| PFL1055c-KpnI-fw | CAGCGGTACCAAACATGGCACAAGAGAGCACAG |
| PFL1055c-AvrII-rv | CAGCCCTAGGATATTTTGAATTATTATTAAGGTCTTCGTC |
| PFL2515c-KpnI-fw | CAGCGGTACCCAAAATGAGTGATCCATGGGCAGATTACGATCC |
| PFL2515c-AvrII-rv | CAGCCCTAGGTGCTCCTGCTCCGTAATATCCATGTTTCTTGATTTTTTTCTC |
| PF07_0010-KpnI-fw | CAGCGGTACCTATAATGAAAGAACAAGAAGAAAGCAACG |
| PF07_0010-AvrII-rv1454 | CAGCCCTAGGGCATCTTCAGCTATTTTATGAAGAG |
| PF08_0004-KpnI-fw | CAGCGGTACCAACTATGAAGAATAAACTTTCTAC |
| PF08_0004-AvrII-rv | CAGCCCTAGGAAGCATCCATACGCGGTTACTTTTTTG |
| PF08_0005-KpnI-fw | CAGCGGTACCCATAATGAACATGTACGTAATCTATTAC |
| PF08_0005-AvrII-rv | CAGCCCTAGGTTGTATTTTTTTAGATATCATAGCTTCTAG |
| PF14_0024-KpnI-fw | CAGCGGTACCTAAAATGTATTCAGGATTTATAAGATTCAGTCAGGC |
| PF14_0024-AvrII-rv | CAGCCCTAGGTTCCAGAAACCAGAACATTGGCCCCTC |
| PF14_0044-KpnI-fw | CAGCGGTACCAAAAATGATAAAAGTACTTCTTGCGGTGC |
| PF14_0044-AvrII-rv | CAGCCCTAGGTTTAGAAAATATAAATGTTATGTTTGC |
| PF14_0046-KpnI-fw | CAGCGGTACCCAAAATGTTGAGTGTTAACAAAGTTACCGC |
| PF14_0046-AvrII-rv | CAGCCCTAGGGAGTTCATCTGAACTGTTAAGTAG |
| PFC1035w-KpnI-fw | CAGCGGTACCTAAAATGTTTATAAAAAGTAGGATAATAAATTTTTATAAG |
| PFC1035w-AvrII-rv | CAGCCCTAGGAATTTCTTTCTTTAATTCAAACATTTCATTATTC |
| PFF0090w-KpnI-fw | CAGCGGTACCCAAAATGACGGACCATTTATTGGATTTTAATATG |
| PFF0090w-AvrII-rv | CAGCCCTAGGATTTTCTGCATTGGCTGAAGCATAAAC |
| PFL0065w-KpnI-fw | CAGCGGTACCCAAAATGAAAACCATAATAATAGTAACCC |
| PFL0065w-AvrII-rv | CAGCCCTAGGTTCTACCATATAAAAATCTGCATC |
| PF13_0191-KpnI-fw | CAGCGGTACCAAGGATGCAAAGTGAATTCTTCATTTGTG |
| PF13_0191-AvrII-rv | CAGCCCTAGGTATGGTATCTAATAAGTTAAGTGTG |
| PF13_0192-KpnI-fw | CAGCGGTACCAAAAATGAAAAGCAAAAAAATAATATGTTCATCTTGC |
| PF13_0192-AvrII-rv | CAGCCCTAGGTAATTTCGTGGGATTTAAAGCTAAGTCC |
| PF13_0192-GST-intern-BamHI-fw | CAGCGGATCCCCTGAGAAGTATTCAAATTATAATAAAAATATACACG |
| PF13_0192-GST-intern-XhoI-rv | CAGCCTCGAGCGATGAATAGGGATCTTTACTTGTTTCTTC |
| PF07_0007-N_mTRAP-KpnI-fw | CAGCGGTACCAAAATGAGTCAACCACAAAAACAACAAAACGAAGAAGGAGCCGCCACTGCCGCCAATACCCAATCTGCATTATATGAACATATGAATAC |
| PF07_0007-Nscr_mTRAP-KpnI-fw | CAGCGGTACCATGGCCAACAATACCCAAACTGAAGCCCAACCACAACAAAAAGCCGAAGCCGGACAAAGTTCTGCATTATATGAACATATGAATAC |
| PF11_0505-N_mTRAP-KpnI-fw | CAGCGGTACCAAAATGGAAGCTGAGAAAAAGGAAGAAAAACAGGAGAAATCCGTGAAAACATTGATGAAGAAATCTGCATTATATGAACATATGAATAC |
| PF11_0505-Nscr_mTRAP-KpnI-fw | CAGCGGTACCATGAAAGAAGAGGAAACATTGGTGGAAAAGAAAAAAGAGAAAGCTATGCAGAAGTCCAAATCTGCATTATATGAACATATGAATAC |
| PFF0090w-N_mTRAP-KpnI-fw | CAGCGGTACCAAAATGACGGACCATTTATTGGATTTTAATATGTATGGATCCCAATTACACAATTTGTTACATTCTGCATTATATGAACATATGAATAC |
| PFF0090w-Nscr_mTRAP-KpnI-fw | CAGCGGTACCATGTTATCCTTTACGCATTTACATATGGGATTGCAAAATCACGACGATAATTATTTATTGTCTGCATTATATGAACATATGAATAC |
| mTRAP-AvrII-rv | CGCGCCTAGGTTCGAGTGCCCAGAATTCTTCTTC |
| mCherry-KpnI-fw | CAGCGGTACCATGGTGAGCAAGGGCGAGGAGGATAAC |
| mCherry-XmaI-rv | CAGCCCCGGGTTACTTGTACAGCTCGTCCATGCCGCCGGTG |
| PF13_0192-XhoI-fw | CAGCCTCGAGAAAAATGAAAAGCAAAAAAATAATATGTTCATCTTGC |
| PF13_0194-XhoI-fw | CAGCCTCGAGTACAATGAAAAGAACAAGACATATTAC |

^*^ Restriction sites are underlined
